# Adipose-derived stem cell exosomes loaded with icariin alleviates rheumatoid arthritis by modulating macrophage polarization in rats

**DOI:** 10.1186/s12951-024-02711-1

**Published:** 2024-07-18

**Authors:** Qiqi Yan, Haixia Liu, Shiyue Sun, Yongsheng Yang, DanPing Fan, Yuqin Yang, Yukun Zhao, Zhiqian Song, Yanjing Chen, Ruyuan Zhu, Zhiguo Zhang

**Affiliations:** 1 Institute of Basic Theory for Chinese Medicine, China Academy of Chinese Medical Sciences, Beijing, China; 2 Institute of Acupuncture and Moxibustion, China Academy of Chinese Medical Sciences, Beijing, China; 3 Institute of Experimental Research Center, China Academy of Chinese Medical Sciences, Beijing, China

**Keywords:** Adipose-derived stem cell exosomes, icariin, Collagen-induced arthritis, Rheumatoid arthritis, Macrophage polarization, Synergistic effect, A novel drug delivery system—adipose-derived stem cells-exosomes (ADSCs-EXO)-ICA

## Abstract

Rheumatoid arthritis (RA) is a chronic autoimmune disease marked by synovitis and cartilage destruction. The active compound, icariin (ICA), derived from the herb Epimedium, exhibits potent anti-inflammatory properties. However, its clinical utility is limited by its water insolubility, poor permeability, and low bioavailability. To address these challenges, we developed a multifunctional drug delivery system—adipose-derived stem cells-exosomes (ADSCs-EXO)-ICA to target active macrophages in synovial tissue and modulate macrophage polarization from M1 to M2. High-performance liquid chromatography analysis confirmed a 92.4 ± 0.008% loading efficiency for ADSCs-EXO-ICA. In vitro studies utilizing cellular immunofluorescence (IF) and flow cytometry demonstrated significant inhibition of M1 macrophage proliferation by ADSCs-EXO-ICA. Enzyme-linked immunosorbent assay, cellular transcriptomics, and real-time quantitative PCR indicated that ADSCs-EXO-ICA promotes an M1-to-M2 phenotypic transition by reducing glycolysis through the inhibition of the ERK/HIF-1α/GLUT1 pathway. In vivo, ADSCs-EXO-ICA effectively accumulated in the joints. Pharmacodynamic assessments revealed that ADSCs-EXO-ICA decreased cytokine levels and mitigated arthritis symptoms in collagen-induced arthritis (CIA) rats. Histological analysis and micro computed tomography confirmed that ADSCs-EXO-ICA markedly ameliorated synovitis and preserved cartilage. Further in vivo studies indicated that ADSCs-EXO-ICA suppresses arthritis by promoting an M1-to-M2 switch and suppressing glycolysis. Western blotting supported the therapeutic efficacy of ADSCs-EXO-ICA in RA, confirming its role in modulating macrophage function through energy metabolism regulation. Thus, this study not only introduces a drug delivery system that significantly enhances the anti-RA efficacy of ADSCs-EXO-ICA but also elucidates its mechanism of action in macrophage function inhibition.

**Graphical abstract:**

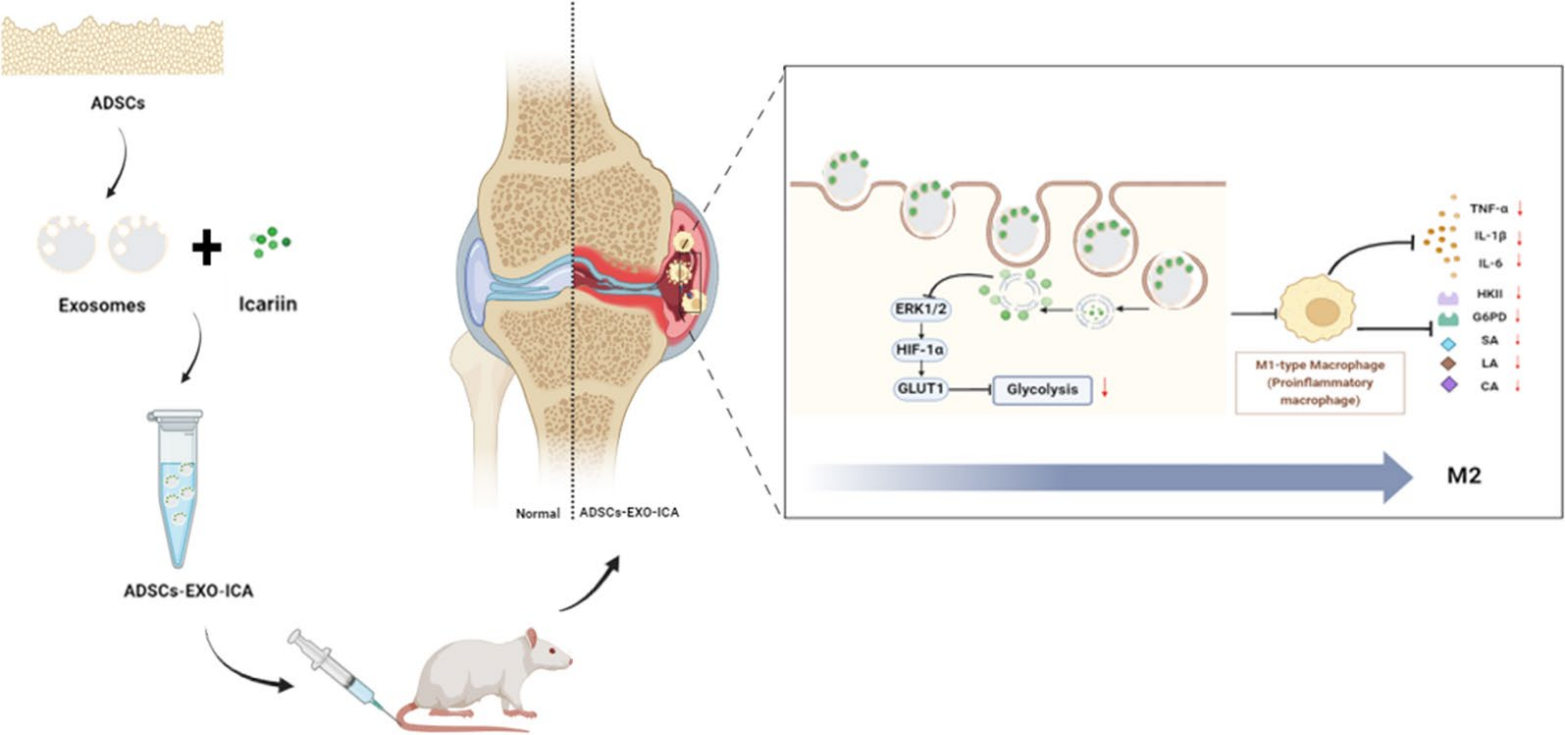

## Introduction

Rheumatoid arthritis (RA) is a chronic autoimmune disease characterized by synovitis and cartilage destruction [1]. The principal etiological factors include an imbalance between pro-inflammatory macrophages (M1-type) and anti-inflammatory macrophages (M2-type) in synovial tissues, which triggers a cascade of inflammatory responses. Macrophages are crucial in the pathogenesis of RA [2], interacting with both non-immune cells (fibroblast-like synoviocytes and chondrocytes) and immune cells (T-cells, neutrophils, and lymphocytes) to exacerbate synovial inflammation and bone erosion [3–5]. An increase in M1-type macrophages in the synovial lining acts as an early indicator of synovitis in RA [6]. These macrophages serve as antigen-presenting cells, initiating T-cell-dependent B-cell activation and the production of pro-inflammatory cytokines. M1-type macrophages produce inflammatory factors, such as tumor necrosis factor (TNF)-α, interleukin (IL)-1β, and IL-6, and express inducible nitric oxide synthase (iNOS). This cascade accelerates bone resorption and destruction, worsening RA progression. Persistent polarization of synovial M1-type macrophages is a key mechanism underlying synovial inflammation and bone destruction in RA. In contrast, M2-type macrophages secrete anti-inflammatory factors, such as IL-10 and transforming growth factor (TGF)-β, and express Arginase 1 (Arg1). Although current treatments for RA are somewhat effective [7, 8], they are not without significant side effects and adverse reactions, highlighting the urgent need for new therapeutic options.

Icariin (ICA), the primary bioactive constituent of the Chinese herb Epimedii Folium, is widely utilized in the treatment of RA and other osteoarticular diseases [9–11]. Extensive research has demonstrated its multifaceted benefits, including the promotion of bone formation, inhibition of bone resorption, and modulation of various signaling pathways [12–16]. Additionally, ICA promotes macrophage polarization to the M2-type by inhibiting the nuclear factor kappa-B (NF-κB) signaling pathway, thereby providing a protective effect [17]. ICA also attenuates particle-induced inflammation and osteolysis by downregulating the NF-κB pathway and inhibiting M1-type polarization [18]. However, its clinical application is constrained by its poor water solubility and low bioavailability.

Adipose-derived mesenchymal stem cells (ADSCs) have advantages, such as easy accessibility, high yield, high proliferation rate, ability to differentiate into different tissues and cells, and strong immunomodulatory ability [19–21]. Exosomes are increasingly utilized for the delivery of small molecule drugs, natural compounds, proteins, and RNAs, benefiting from their non-immunogenic, non-infusion toxic properties, and lack of tumorigenic potential [22]. Adipose-derived stem cell exosomes (ADSCs-EXO) are more stable, smaller and less likely to provoke an immune response than ADSCs [19, 23]. Recent studies have explored using milk exosomes and bone marrow mesenchymal stem cell exosomes (BMSCs-EXO) loaded with ICA to facilitate the repair of cartilage and bone damage [13, 14]. Over the past decades, there has been novel evidence that exosomes are effectual players in the pathological mechanisms of autoimmune diseases such as RA [24, 25]. While some studies have noted the anti-inflammatory and immunomodulatory effects of ADSCs-EXO in treating RA by modulating macrophage polarization [26], the use of ADSCs-EXO loaded with ICA for RA treatment through macrophage polarization modulation has not yet been documented.

M1-type macrophages primarily rely on glycolysis for energy metabolism, ensuring a rapid energy supply [27]. Activated M1-type macrophages can increase the levels of intermediate metabolites such as succinic acid (SA), citric acid (CA), and lactic acid (LA), which are crucial components of the tricarboxylic acid cycle [27]. Enhanced glycolysis and mitochondrial rupture lead to the reprogramming of glucose metabolism in macrophages, shifting them from a resting state to the pro-inflammatory M1-type and triggering an intensified inflammatory response [25]. Furthermore, macrophage glycolysis increases intermediate metabolites, upregulates glycolytic enzymes such as hexokinase (HKII) and glucose-6-phosphate dehydrogenase (G6PD), and promotes inflammatory responses [28]. In RA, the ERK/HIF-1α/GLUT1 signaling pathway appears to play a crucial role in the polarization of M1-type macrophages [23, 29]. Increased intermediate metabolites like SA and CA stabilize HIF-1α, enhancing the transcriptional expression of M1-type macrophage effector molecules [30]. Elevated HIF-1α not only increases the transcription of genes related to the macrophage glycolysis pathway (GLUT1) but also regulates enzymes necessary for macrophage glycolysis, such as lactate dehydrogenase A (LDHA), hexokinase type II (HKII), and pyruvate kinase isozyme typeM2 (PKM2), which promote macrophage polarization towards the M1-type and thus exacerbate RA [31].

**Fig. 1:**
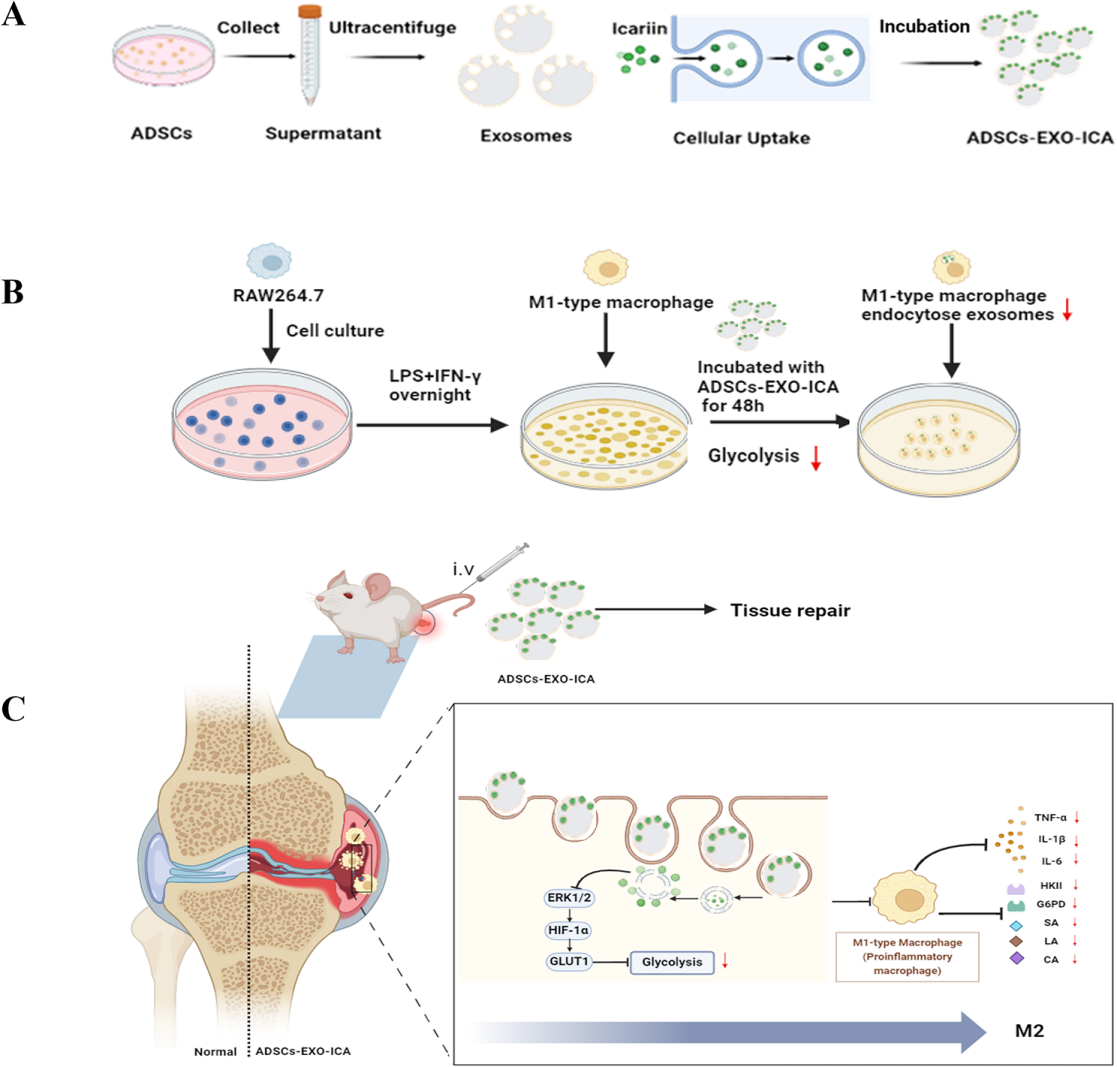
(**A-C**) The drug delivery system—adipose-derived stem cell exosomes (ADSCs-EXO) loaded with icariin (ICA)—synergistically inhibits M1-type macrophage inflammation induced by lipopolysaccharide (LPS) and interferon-gamma, as well as the arthritic symptoms in collagen-induced arthritis (CIA) rats. ADSCs-EXO-ICA adjust macrophage polarization by inhibiting glycolysis mediated by the ERK1/2/HIF-1α/GLUT1 pathway

In summary, both ADSCs-EXO and ICA have the capability to modulate macrophage polarization. However, it is unclear whether these two agents synergistically contribute to inhibiting macrophage glycolysis and regulating macrophage glycolytic reprogramming during the RA process. Consequently, we have developed a multifunctional drug delivery system, ADSCs-EXO-ICA, to co-deliver drugs that modulate macrophage polarization and validate drug efficacy in RA (Fig. 1A and B, and 1C).

## Results

### Preparation and characterization of ADSCs-EXO and ADSCs-EXO-ICA

**Fig. 2:**
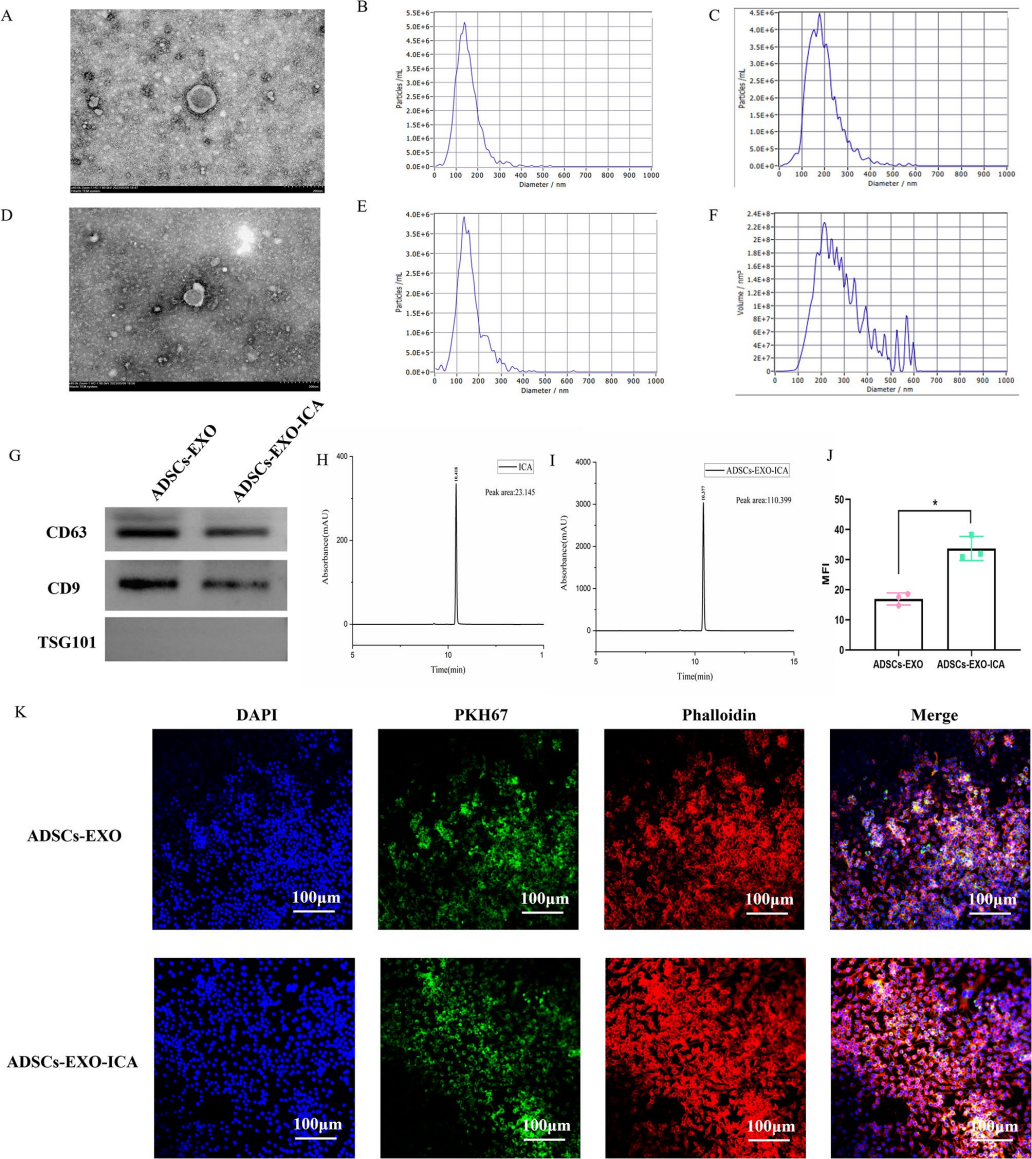
Characterization of ADSCs-EXO and ADSCs-EXO-ICA using TEM, NTA, and Western Blot. (**A**,** D**) TEM images of ADSCs-EXO and ADSCs-EXO-ICA. (**B**,** C**,** E**, and **F**) NTA characterization of ADSCs-EXO and ADSCs-EXO-ICA. (**G**) Western Blot analysis of ADSCs-EXO and ADSCs-EXO-ICA. (**H**,** I**) Encapsulation rate of ADSCs-EXO-ICA determined by HPLC. (**J**) Quantitative analysis of the average fluorescence intensity for both ADSCs-EXO and ADSCs-EXO-ICA. Data were expressed as mean ± SD (*n* = 3, *p < 0.05 vs. the ADSCs-EXO group). (**K**) Immunofluorescence imaging of PKH-67-labeled ADSCs-EXO and ADSCs-EXO-ICA

ADSCs-EXO, extracted by ultracentrifugation, exhibited characteristic cup-shaped morphology as observed by TEM (Fig. 2A). The particle size measured by NTA was approximately 134.5 nm, with a concentration of 3.9E + 6 particles/mL (Fig. 2B-C), consistent with typical exosome dimensions. Western blot (WB) analysis confirmed the presence of exosome markers TSG101, CD9, and CD63, validating the successful isolation of ADSCs-EXO (Fig. 2G).

Furthermore, ADSCs-EXO-ICA were characterized using TEM, NTA, and WB. TEM analysis indicated that ADSCs-EXO-ICA maintained the typical lipid bilayer structure, suggesting that the exosome structure was intact post-drug loading (Fig. 2D). NTA results showed that drug loading had a minimal impact on the particle size of ADSCs-EXO-ICA, which measured approximately 177.6 nm (Fig. 2E-F). WB results demonstrated that the biological functions of the exosomes were preserved following drug incorporation (Fig. 2G).

ADSCs-EXO-ICA was prepared by incubating ADSCs-EXO with ICA at a 1:1 ratio (w/w), achieving a maximum encapsulation rate of 92.4 ± 0.008% as determined by HPLC (Fig. 2H-I). Additionally, we labeled ADSCs-EXO with PKH67 and co-incubated these labeled exosomes with M1-type macrophages for 8 h to assess cellular uptake. The results indicated that the mean fluorescence intensity (MFI) in the ADSCs-EXO-ICA group was higher than that in the ADSCs-EXO group (Fig. 2J and K), confirming increased cellular uptake efficiency of ADSCs-EXO-ICA by M1-type macrophages following loading with ICA.

### ADSCs-EXO-ICA modulation of macrophage polarization and inflammation in vitro

**Fig. 3:**
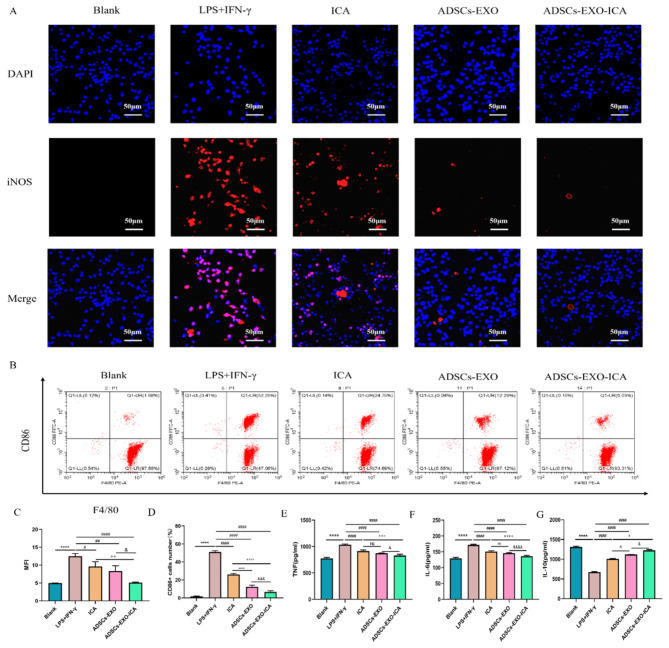
ICA, ADSCs-EXO, and ADSCs-EXO-ICA reduce macrophage polarization towards the M1-type, particularly ADSCs-EXO-ICA. (**A**) Expression of iNOS in LPS + IFN-γ-induced macrophages treated with ICA, ADSCs-EXO, and ADSCs-EXO-ICA. (**B**) Percentage of M1 macrophages (F4/80^+^CD86^+^) treated with the interventions. (**C**) Immunofluorescence results and quantitative analysis of M1-type with different interventions. (**D**) Flow cytometry results and qualitative analysis of M1-type with different interventions. (**E-G**) Levels of cytokines (TNF-α, IL-6, and IL-10) in the cell supernatant. Data were expressed as mean ± SD (*n* = 3, **** *p* < 0.0001 vs. the Blank group, ^#^*p* < 0.05, ^###^*p* < 0.001 and ^####^*p* < 0.0001 vs. the LPS + IFN-γ group, ^&^*p* < 0.05, ^&&&^*p* < 0.001, and ^&&&&^*p* < 0.0001 vs. the ADSCs-EXO group, ^++^*p* < 0.01, ^+++^*p* < 0.001, and ^++++^*p* < 0.0001 vs. the ICA group, ^^^^*p* < 0.01 vs. the ICA group, MFI represented mean fluorescence intensity. Blue = DAPI channel, red = iNOS channel. Scale bar = 50 μm)

iNOS (M1 marker) and CD86 (M1 marker) were analyzed using immunofluorescence (Fig. 3A and C) and flow cytometry (Fig. 3B and D) to assess the anti-inflammatory effects of ICA, ADSCs-EXO, and ADSCs-EXO-ICA on M1-type macrophages. Compared to the LPS + IFN-γ group, the expression of iNOS and the number of M1-type macrophages (F4/80 + CD86+) were significantly reduced by ICA, ADSCs-EXO, and ADSCs-EXO-ICA, indicating that ADSCs-EXO-ICA may inhibit the polarization of macrophages towards the M1 phenotype. Furthermore, ADSCs-EXO-ICA more effectively suppressed iNOS expression and the percentage of M1-type macrophages than either ICA or ADSCs-EXO alone. These results suggest that ADSCs-EXO-ICA can effectively inhibit the inflammatory response in M1-type macrophages induced by LPS + IFN-γ, demonstrating the benefits of synergistic drug delivery.

Subsequently, we measured the levels of inflammatory cytokines (TNF-α and IL-6) and an anti-inflammatory cytokine (IL-10) in the cell supernatants following treatment with the different formulations (Fig. 3E-F). The LPS + IFN-γ group showed significantly increased secretion of TNF-α and IL-6 and decreased levels of IL-10 compared to the Blank group. In contrast, the groups treated with ICA, ADSCs-EXO, and ADSCs-EXO-ICA significantly reduced the levels of TNF-α and IL-6 and increased IL-10 levels, particularly in the ADSCs-EXO-ICA group. Corresponding to the effects on M1-type macrophages, ADSCs-EXO-ICA was more effective in reducing inflammatory cytokines and enhancing IL-10 levels than ICA or ADSCs-EXO alone. These findings indicate that ADSCs-EXO-ICA can inhibit the secretion of pro-inflammatory factors and promote the secretion of anti-inflammatory factors by macrophages in vitro (Fig. 3G).

### ADSCs-EXO-ICA modulates macrophage polarization via the ERK1/2/HIF-1a/GLUT1 pathway in vitro

**Fig. 4:**
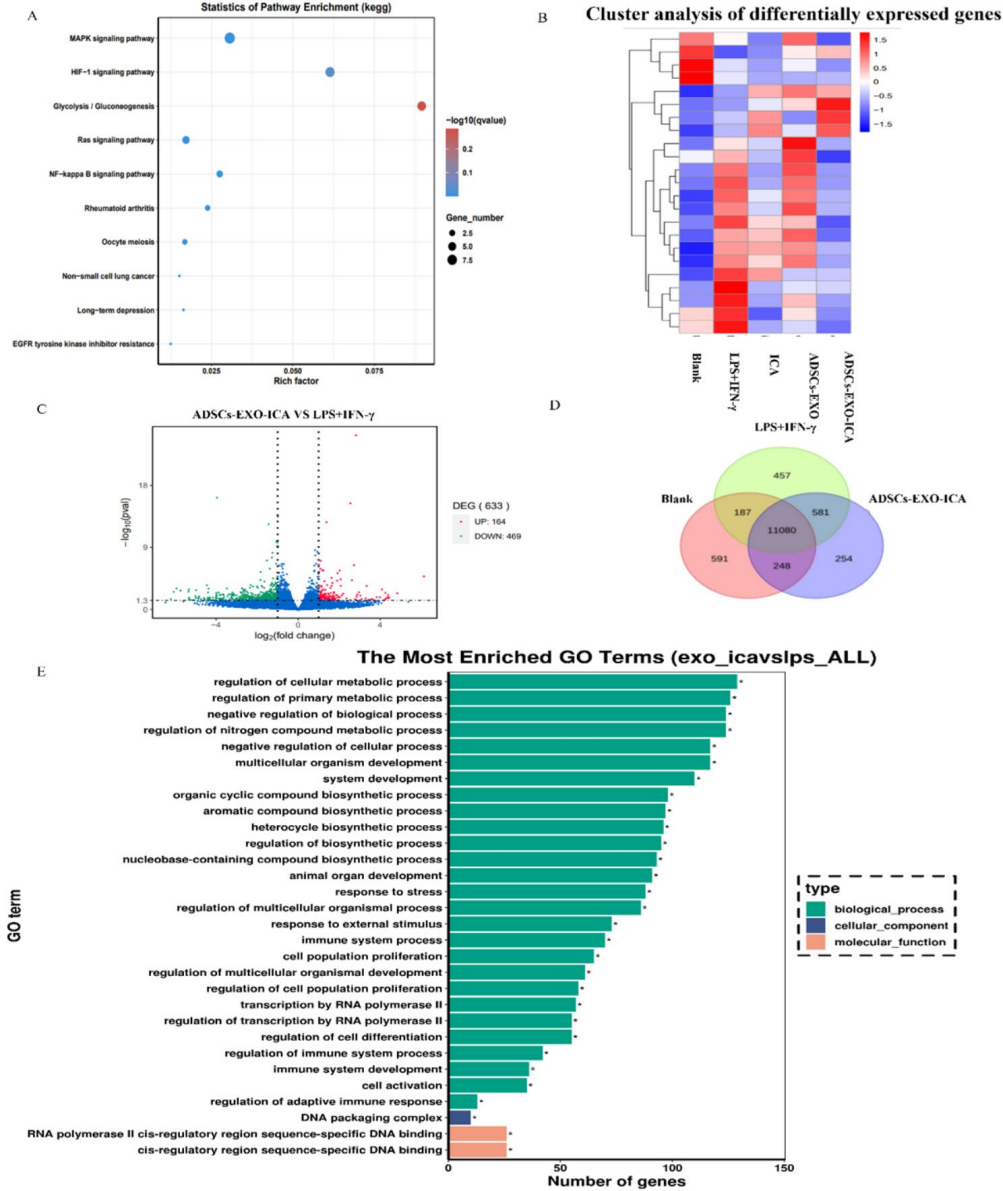
Molecular mechanisms of ADSCs-EXO-ICA. (**A**) KEGG pathway analysis of activated RAW264.7 macrophages treated with ICA, ADSCs-EXO, and ADSCs-EXO-ICA. (**B**) Differential gene clustering diagram of ERK1/2, HIF-1α and GLUT1. (**C**) Differential Gene Volcano diagram. (**D**) Differential Gene Venn diagram. (**E**) GO enrichment histogram

The molecular mechanisms underlying the anti-inflammatory response of ADSCs-EXO-ICA were explored through RNA sequencing, revealing 11,661 intersecting potential targets related to the treatment of the M1-type macrophage inflammatory response (Fig. 4D). The volcano plot indicated that ADSCs-EXO-ICA upregulated 164 genes and downregulated 469 genes in M1-type macrophages (Fig. 4C). Differential gene clustering analysis suggested that the ERK1/2 cascade and hypoxic processes are closely linked with ADSCs-EXO-ICA’s ability to mitigate inflammation induced by M1 macrophages (Fig. 4B). HIF-1α, a critical factor in hypoxic processes [32], and ERK1/2, a key member of the MAPK family [33, 34], are known to regulate the expression of HIF-1α [35]. Subsequent GO enrichment analysis for biological processes (BP), molecular functions (MF), and cellular components (CC) identified the top three BPs as regulation of primary metabolic processes and negative regulation of biological processes. These results indicate that ADSCs-EXO-ICA can modulate the metabolic processes associated with M1-type macrophages (Fig. 4E). KEGG analysis further suggested that the molecular mechanisms of ADSCs-EXO-ICA are linked to the MAPK, HIF-1α, and glycolysis signaling pathways (Fig. 4A). GLUT1, involved in glucose metabolism and the immune response [36, 37], is regulated by HIF-1α, which in turn can inhibit glycolysis [38]. Thus, the ERK1/2/HIF-1a/GLUT1 pathway is significantly influenced by ADSCs-EXO-ICA.

**Fig. 5:**
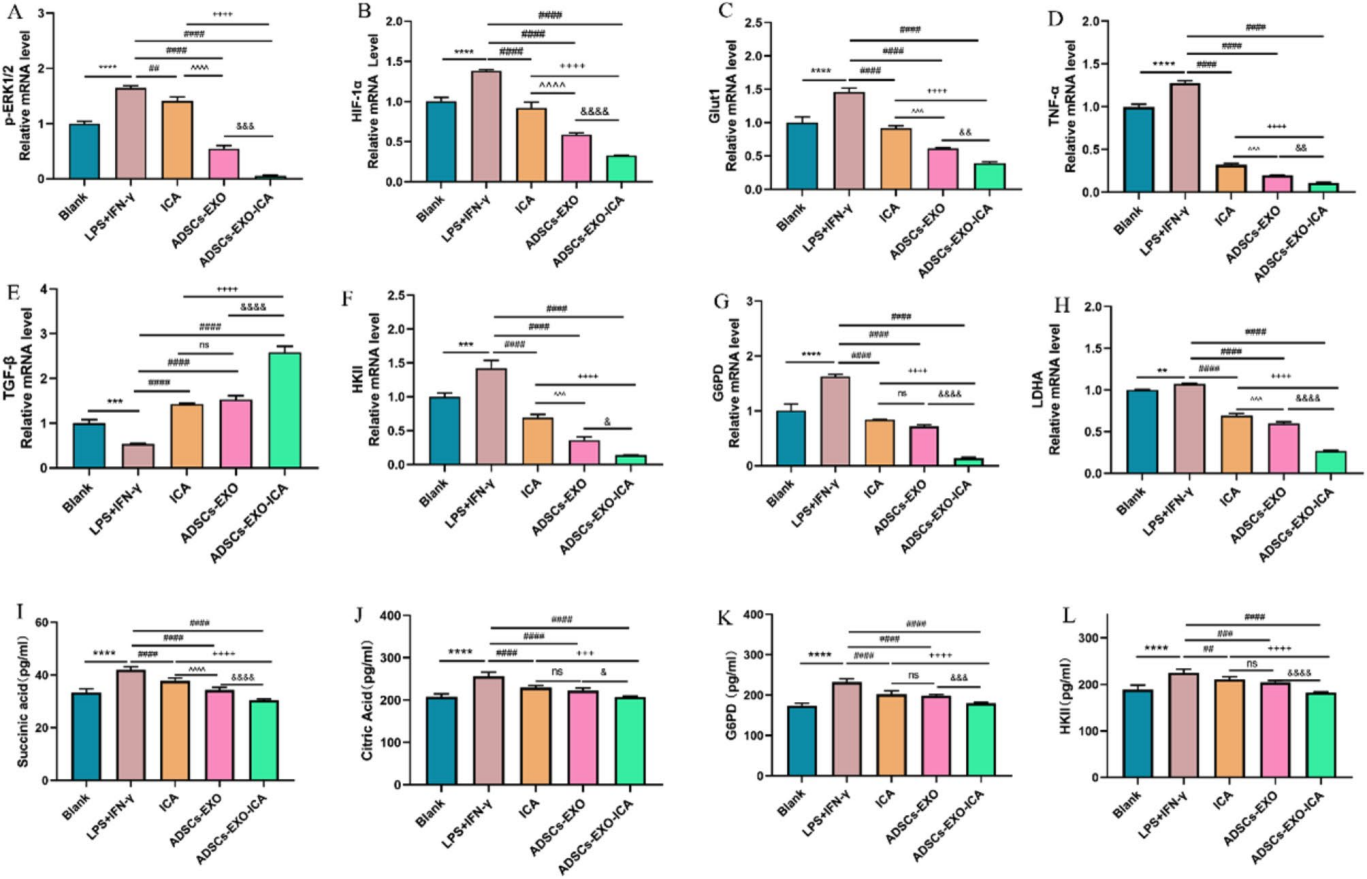
(**A-C**) Relative mRNA expression levels of Glut1, HIF-1α, and ERK1/2 in activated RAW264.7 macrophages incubated with ADSCs-EXO-ICA detected by quantitative real-time PCR (qRT-PCR). (**D-E**) Relative mRNA expression of TNF-α (marked M1-type macrophage) and TGF-β (marked M2-type macrophage) examined by qRT-PCR. (**F-H**) Relative mRNA expression of HKII, G6PD and LDHA examined by qRT-PCR. (**J-L**) Levels of Succinic acid, Citric acid, G6PD and HKII in cell supernatant. Datas were expressed as mean ± SD (*n* = 3, ** *p* < 0.01 *** *p* < 0.001 and **** *p* < 0.0001 vs. the Blank group, ^# #^*p* < 0.01, ^###^*p* < 0.001 and ^####^*p* < 0.0001 vs. the LPS + IFN-γ group, ^&^*p* < 0.05, ^&&^*p* < 0.01, ^&&&^*p* < 0.001, and ^&&&&^*p* < 0.0001 vs. the ADSCs-EXO group, ^+++^*p* < 0.001, and ^++++^*p* < 0.0001 vs. the ICA group, ^^^^*p* < 0.01 and ^^^^^^*p* < 0.0001 vs. the ICA group)

Therefore, we examined the expression of p-ERK1/2, HIF-1α, glycolysis-related mRNA (GLUT1, HKII, G6PD, and LDHA), TNF-α (M1-type marker), and TGF-β (M2-type marker) in vitro using RT-qPCR. The LPS + IFN-γ group showed a significant increase in the expression of p-ERK1/2 (Fig. 5A), HIF-1α (Fig. 5B), glycolysis-related mRNA (GLUT1, HKII, G6PD, and LDHA) (Fig. 5C and F-H), and TNF-α, and a decrease in TGF-β expression compared with the blank group. However, the groups treated with ICA, ADSCs-EXO, and ADSCs-EXO-ICA significantly reduced the expression of p-ERK1/2, HIF-1α, glycolysis-related mRNA, and TNF-α, and increased the expression of TGF-β, particularly in the ADSCs-EXO-ICA group. Furthermore, we assessed the levels of glycolysis-related intermediary metabolites and enzymes (Succinic acid, Citric acid, G6PD, and HKII) in the cellular supernatant by ELISA. The LPS + IFN-γ group significantly increased the levels of Succinic acid, Citric acid, G6PD, and HKII compared with the blank group (Fig. 5I-L). Conversely, the groups of ICA, ADSCs-EXO, and ADSCs-EXO-ICA significantly lowered the levels of these substances compared with the LPS + IFN-γ group. ADSCs-EXO-ICA more effectively mitigated glycolysis levels than either ADSCs-EXO or ICA alone. These results further indicate that the ability of ADSCs-EXO-ICA to modulate macrophage polarization is closely linked to the ERK1/2/HIF-1α/GLUT1 pathway.

### Biodistribution in CIA rats

**Fig. 6:**
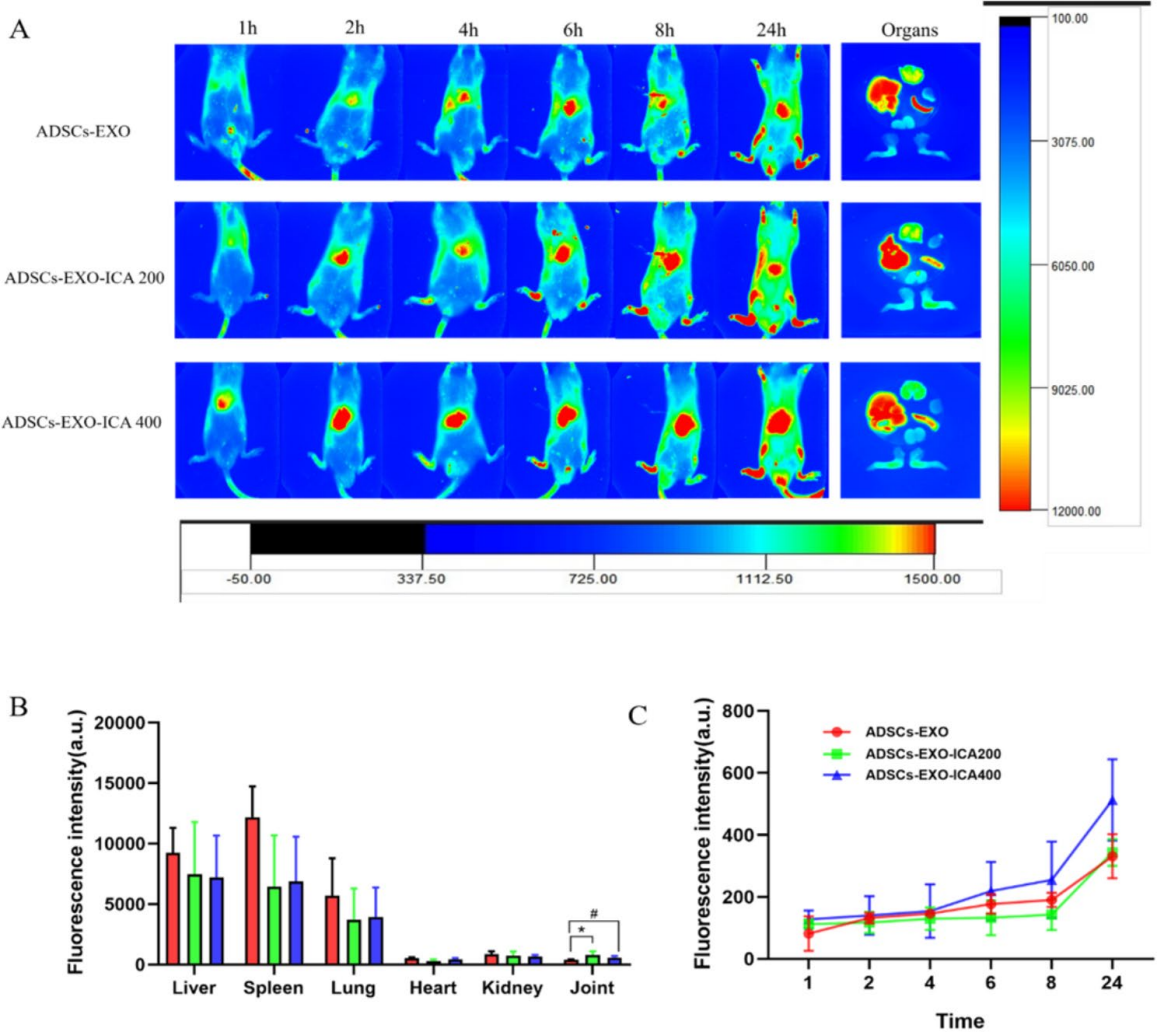
Biodistribution and therapeutic treatment of ADSCs-EXO, ADSCs-EXO-ICA200, and ADSCs-EXO-ICA400 in CIA rats. (**A**) Real-time fluorescence imaging of CIA rats in vivo and ex vivo imaging of organs 24 h after intravenous injection of ADSCs-EXO, ADSCs-EXO-ICA200, and ADSCs-EXO-ICA400 (*n* = 3). (**B**) Semi-quantitation of fluorescence intensity in joints and organs. (**C**) Fluorescence intensity in CIA rats 24 h post-intravenous injection of the respective treatments. (Datas were expressed as mean ± SD, *n* = 3, ^*^p < 0.05 vs. the ADSCs-EXO, ^##^*p* < 0.01 vs. the ADSCs-EXO group)

CIA rats were intravenously injected through the tail vein with ADSCs-EXO, ADSCs-EXO-ICA200, or ADSCs-EXO-ICA400. Over time, the fluorescence intensity at the ankle joint of ADSCs-EXO, ADSCs-EXO-ICA200, and ADSCs-EXO-ICA400 was observed to increase progressively (Fig. 6A-C). Notably, the fluorescence intensity of ADSCs-EXO-ICA200 and ADSCs-EXO-ICA400 was significantly higher than that of ADSCs-EXO, indicating enhanced accumulation of ADSCs-EXO-ICA at the ankle joint (Fig. 6B).

To assess the distribution of ADSCs-EXO-ICA in various organs of CIA rats, the liver, kidney, spleen, heart, and lungs, as well as ankle joints, were analyzed 24 h post-injection. The fluorescence intensity in the liver and spleen was notably higher for all groups, suggesting that ADSCs-EXO-ICA primarily localizes to these organs (Fig. 6B).

### Therapeutic efficacy of ADSCs-EXO-ICA200 in early arthritis in CIA rats

**Fig. 7:**
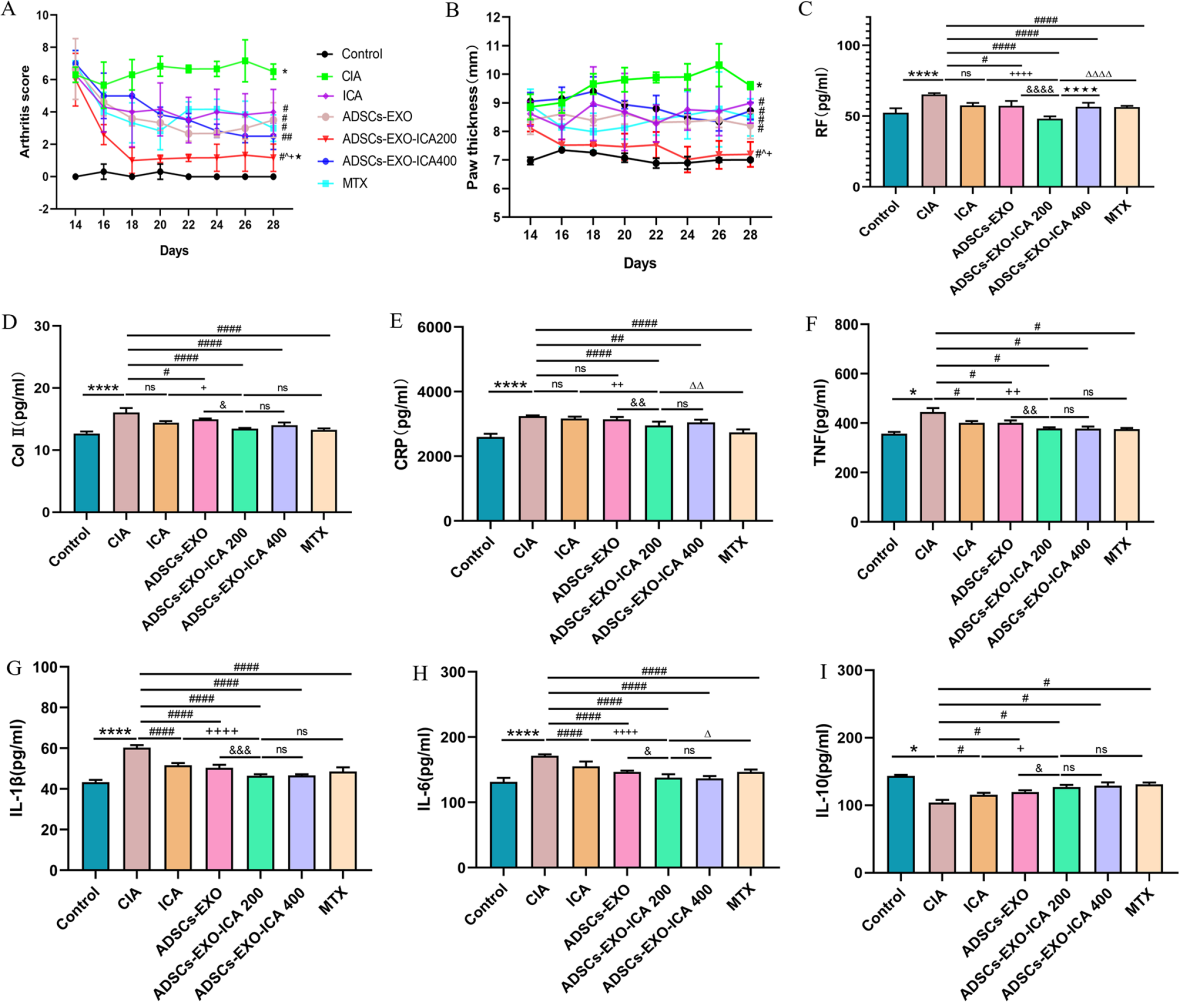
ADSCs-EXO-ICA demonstrates superior anti-inflammatory efficacy in early arthritis in CIA rats. (**A**) Arthritis score in different treatment groups over 14 days (*n* = 5). (**B**) Paw thickness in different groups over 14 days. (**C-D**) Levels of Rheumatoid Factor (RF) and Collagen II (CII) in CIA rats. (E-H) Cytokine levels (CRP, TNF-α, IL-1β, IL-6, and IL-10) in the serum of CIA rats. (Data were expressed as mean ± SD (*n* = 3, ^*^p < 0.05, ^**^p < 0.01 and ^****^p < 0.0001 vs. the Control group, ^#^*p* < 0.05, ^##^*p* < 0.01, ^###^*p* < 0.001 and ^####^*p* < 0.0001 vs. the CIA group, ^&^*p* < 0.05, ^&&^*p* < 0.01, ^&&&^*p* < 0.001,and ^&&&&^*p* < 0.0001 vs. the ADSCs-EXO group, ^+^*p* < 0.05, ^++^*p* < 0.01, ^+++^*p* < 0.001and ^++++^*p* < 0.0001 vs. the ICA group, •• *p* < 0.01 and •••• *p* < 0.0001 vs. the ADSCs-EXO-ICA400 group, ^Δ^*p* < 0.05 and ^ΔΔΔΔ^*p* < 0.0001 vs. the MTX group, ns = no significance)

The severity of arthritis in CIA rats was evaluated using the Arthritis Index (AI) and paw thickness measurements (Fig. 7A-B). One week after the second immunization, both AI and paw thickness significantly increased in the CIA group. Treatments with ICA, ADSCs-EXO, ADSCs-EXO-ICA200, ADSCs-EXO-ICA400, and MTX significantly alleviated these arthritis symptoms, with ADSCs-EXO-ICA200 showing particularly notable effects. After two weeks of treatment, both AI and paw thickness were significantly lower in the ADSCs-EXO-ICA200 group compared to the ICA or ADSCs-EXO groups, demonstrating a more pronounced ameliorative impact on arthritic symptoms than ICA or ADSCs-EXO alone.

To assess joint inflammation, levels of inflammatory cytokines (CRP, TNF-α, IL-1β, and IL-6) secreted by M1-type macrophages, along with arthritic indicators (Rheumatoid Factor (RF) and Collagen II (CII)), were measured in serum samples from CIA rats using ELISA (Fig. 7C-H). One week after the second immunization, these indicators were significantly elevated in the CIA group compared to the normal group. However, the levels were significantly reduced in the groups treated with ICA, ADSCs-EXO, ADSCs-EXO-ICA200, ADSCs-EXO-ICA400, and MTX, particularly in the ADSCs-EXO-ICA200 group. Compared to the ICA, ADSCs-EXO, ADSCs-EXO-ICA400, and MTX groups, the ADSCs-EXO-ICA200 group exhibited more pronounced reductions in these indicators. The anti-inflammatory effects were comparable to those observed in the MTX group. Above results indicated that ADSCs-EXO-ICA200 significantly ameliorated the inflammatory and glycolysis microenvironment of the ankle joints more effectively than other treatments. Conversely, levels of the anti-inflammatory cytokine IL-10, secreted by M2-type macrophages, were inversely related to the aforementioned inflammatory cytokines, and the anti-inflammatory effects of ADSCs-EXO-ICA surpassed those of the single treatment groups (Fig. 7I). These results suggest that ADSCs-EXO-ICA200 can modulate macrophage polarization, thereby alleviating the severity of synovitis.

**Fig. 8:**
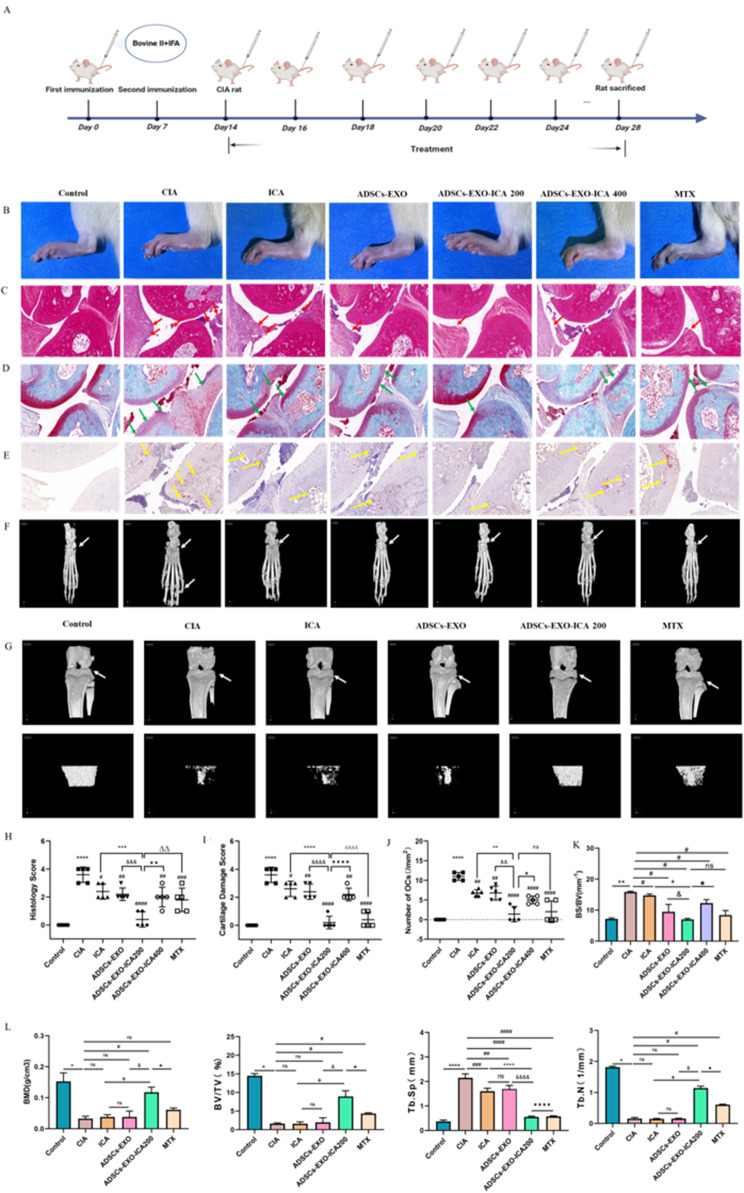
Therapeutic efficacy of different treatments, especially ADSCs-EXO-ICA200. (**A**) Study procedure for a therapeutic regimen in a CIA rat model. (**B**) Exterior images of rats receiving different treatments. (**C**,** H**) Representative Hematoxylin-eosin (**H**,** E**) staining of ankle joint samples from different drug groups in histological analysis. Red arrows indicate the synovium. (**D**,** I**) Representative Safranin O-Fast green (S-O) staining of ankle joint samples from different drug groups in histological analysis. Green arrows point to articular cartilage. (**E**,** J**) Representative tartrate-resistant acid phosphatase (TRAP) staining of ankle joint samples from different drug groups in histological analysis. Yellow arrows to osteoclasts (scale bars: H&E and S-O = 200 μm, TRAP = 100 μm). (**F-K**) Representative micro-CT images and quantitative analyses of ankle joints from rats with different treatments. (**G-L**) Representative micro-CT images and quantitative analyses of knee joints from rats with different treatments. (*n* = 3, BS/BV, BMD, BV/TV, Tb.Sp, Tb.N). Data were expressed as mean ± SD (*n* = 3, ^*^p < 0.05, ^**^p < 0.01 and ^****^p < 0.0001 vs. the Control group, ^#^*p* < 0.05, ^##^*p* < 0.01, ^###^*p* < 0.001 and ^####^*p* < 0.0001 vs. the CIA group, ^&^*p* < 0.05, ^&&^*p* < 0.01, ^&&&^*p* < 0.001,and ^&&&&^*p* < 0.0001 vs. the ADSCs-EXO group, ^+^*p* < 0.05, ^++^*p* < 0.01, ^+++^*p* < 0.001, and ^++++^*p* < 0.0001 vs. the ICA group, • *p* < 0.05, •• *p* < 0.01 •••• *p* < 0.0001 vs. the ADSCs-EXO-ICA200 group, ^ΔΔ^*p* < 0.01 and ^ΔΔΔΔ^*p* < 0.0001 vs. the ADSCs-EXO-ICA200 group, ns = no significance)

Given the excellent anti-inflammatory properties and targeted delivery to the ankle of ADSCs-EXO-ICA, we investigated its efficacy in CIA rats (Fig. 8A). As shown in Fig. 8B, the paw exterior images of each group of rats after various drug treatments displayed noticeable differences. To further assess the therapeutic efficacy of ICA, ADSCs-EXO, ADSCs-EXO-ICA200, ADSCs-EXO-ICA400, and MTX on the inflammation of ankle joints in CIA rats, we examined the histopathological changes in the ankle joints (Fig. 8C-E and H-J). The ankle joints in the normal group exhibited normal histology with no synovial hyperplasia or inflammatory cell infiltration (HE staining), smooth and even articular cartilage surfaces (Safranin O-fast green (S-O) staining), and a minimal presence of osteoclasts (TRAP staining). However, two weeks after the second immunization, the ankle joints of CIA rats showed pronounced pathological changes including synovial hyperplasia with vascular opacities, inflammatory cell infiltration, uneven articular cartilage surfaces, and an increased number of osteoclasts compared to the normal group. These pathological changes were significantly mitigated by treatments with ICA, ADSCs-EXO, ADSCs-EXO-ICA200, ADSCs-EXO-ICA400, and MTX, particularly with ADSCs-EXO-ICA200. Notably, ADSCs-EXO-ICA200 more effectively alleviated the histopathological changes in the ankle joints than either ICA or ADSCs-EXO alone, was superior to ADSCs-EXO-ICA400, and comparable to MTX. These results suggest that ADSCs-EXO-ICA200 offers a significant protective effect against arthritis in CIA rats, likely due to the benefits of synergistic drug delivery.

To assess the impact of ICA, ADSCs-EXO, ADSCs-EXO-ICA200, ADSCs-EXO-ICA400, and MTX on the bone microstructure of CIA rats, we utilized micro-CT to examine changes in bone surface to volume ratio (BS/BV) at the ankle joints of the various treatment groups (Fig. 8F-K). Compared to the CIA group, all treatments, especially ADSCs-EXO-ICA200, significantly reduced BS/BV. Notably, ADSCs-EXO-ICA200 more effectively improved the bone microstructure of the ankle joints than either ICA or ADSCs-EXO alone, was superior to ADSCs-EXO-ICA400, and comparable with MTX. These results indicate that ADSCs-EXO-ICA200 markedly improves the bone microstructure and provides protection to the ankle joints. Additionally, compared to the CIA group, ADSCs-EXO-ICA200 significantly enhanced bone mineral density (BMD), bone volume to total volume ratio (BV/TV), trabecular number (Tb.N), and reduced trabecular separation (Tb.Sp) at the knee joint. In comparison with the MTX group, ADSCs-EXO-ICA200 more effectively promoted BV/TV, Tb.N, and reduced Tb.Sp at the knee joint, suggesting that ADSCs-EXO-ICA200 better supports trabecular formation and BMD, and protects the bone tissue of the knee joints (Fig. 8G-L).

### ADSCs-EXO-ICA200 modulates M1-to-M2 phenotypic switch by inhibiting glycolysis through the ERK/HIF-1α/GLUT1 pathway in vivo

**Fig. 9:**
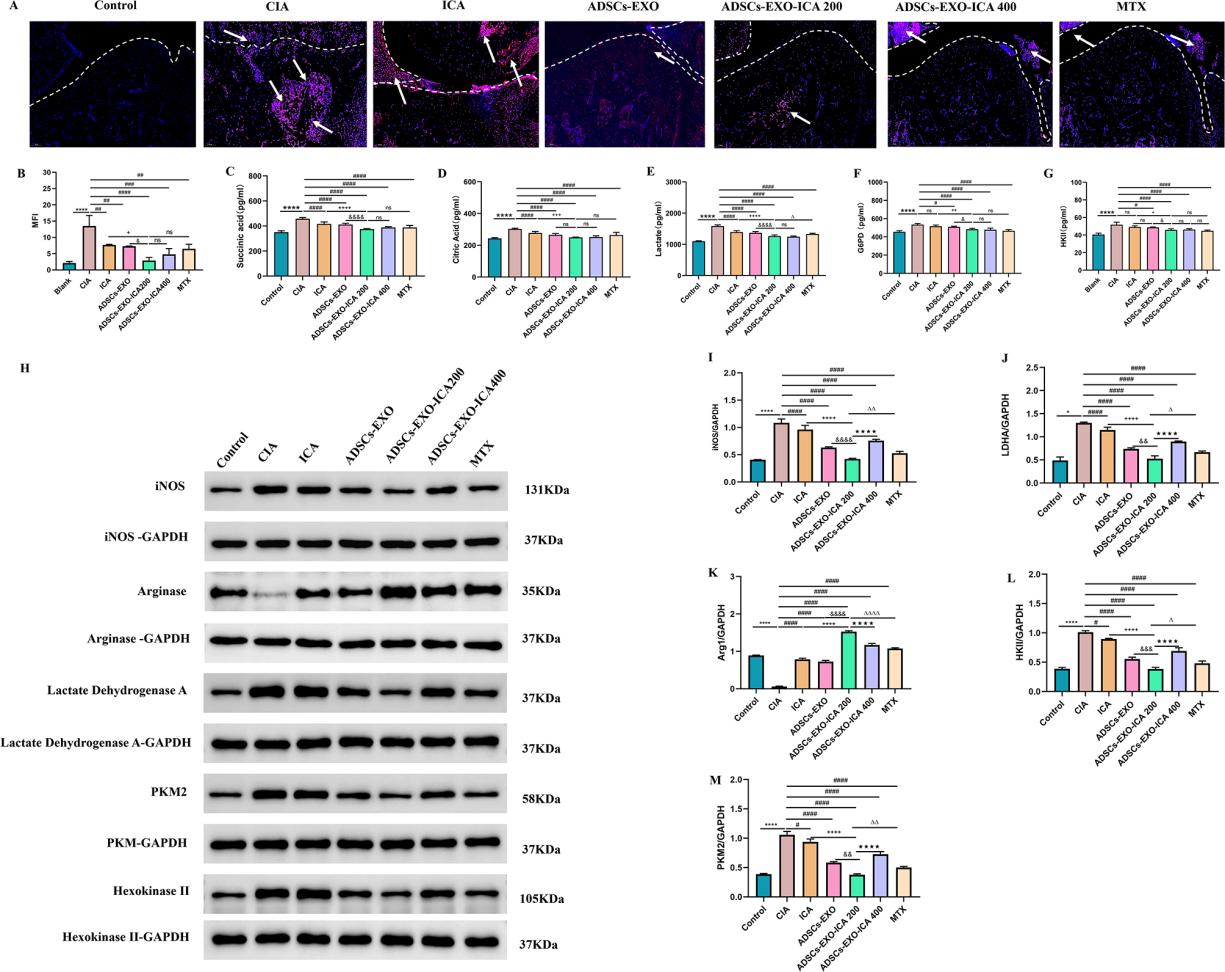
ADSCs-EXO-ICA inhibited early arthritis in CIA rats, potentially by inhibiting glycolysis in macrophages, thus reducing M1-type macrophage polarization. (**A**) Representative images and immunofluorescence analysis of iNOS expression in ankle joint samples with different treatments (white arrows indicate iNOS-labeled M1-type macrophages, scale bar = 100 μm). (**B-G**) Levels of glycolysis indicators (SA, CA, LDHA, HKII, G6PD, ATP, and glucose consumption) in serum from CIA rats. (**H**) WB images and (**I-M**) relative protein expression of iNOS, Arg1, LDHA, PKM2, and HKII. Data were expressed as mean ± SD (*n* = 3, ^*^p < 0.05 and ^****^p < 0.0001 vs. the Control group, ^#^*p* < 0.05, ^##^*p* < 0.01, ^###^*p* < 0.001 and ^####^*p* < 0.0001 vs. the CIA group, ^&^*p* < 0.05, ^&&^*p* < 0.01, ^&&&^*p* < 0.001,and ^&&&&^*p* < 0.0001 vs. the ADSCs-EXO group, ^+^*p* < 0.05, ^++^*p* < 0.01, ^+++^*p* < 0.001, and ^++++^*p* < 0.0001 vs. the ICA group, •••• *p* < 0.0001 vs. the ADSCs-EXO-ICA200 group, ^Δ^*p* < 0.05, ^ΔΔ^*p* < 0.01 and ^ΔΔΔΔ^*p* < 0.0001 vs. the ADSCs-EXO-ICA200 group, ns = no significance)

To explore the relationship between ADSCs-EXO-ICA and macrophage polarization in CIA rats, the expression of iNOS-labeled M1-type macrophages in ankle joints was analyzed using immunofluorescence (Fig. 9A-B) and Western Blot (WB) (Fig. 9H-I). Compared with the CIA group, treatments with ICA, ADSCs-EXO, ADSCs-EXO-ICA200, ADSCs-EXO-ICA400, and MTX significantly reduced both the expression of iNOS-marked M1-type macrophages and iNOS protein levels. Notably, ADSCs-EXO-ICA200 demonstrated a more pronounced inhibitory effect on iNOS-marked M1-type macrophages and iNOS protein levels than either ICA or ADSCs-EXO alone. Conversely, the protein levels of Arg1-marked M2-type macrophages in synovial tissue showed an inverse pattern to iNOS (Fig. 9H-K). These findings suggest that ADSCs-EXO-ICA200 modulates inflammation by suppressing M1-type macrophages and promoting M2-type macrophage polarization.

M1-type macrophage polarization is associated with increased glycolytic activity in joint tissues. Consequently, the levels of glycolysis indicators (SA, CA, LDHA, HKII, G6PD) in the serum of CIA rats were measured by ELISA (Fig. 9C-G), and the protein expression of glycolysis indicators (LDHA, PKM2, HKII) in synovial tissue was assessed by WB (Fig. 9G and M, and Fig. 9L). Correspondingly, these glycolysis indicators in the serum, related to M1-type macrophage activity, displayed similar trends to the protein expression of iNOS. These results indicate that ADSCs-EXO-ICA200 alleviates joint inflammation by inhibiting glycolysis in M1-type macrophages.

**Fig. 10:**
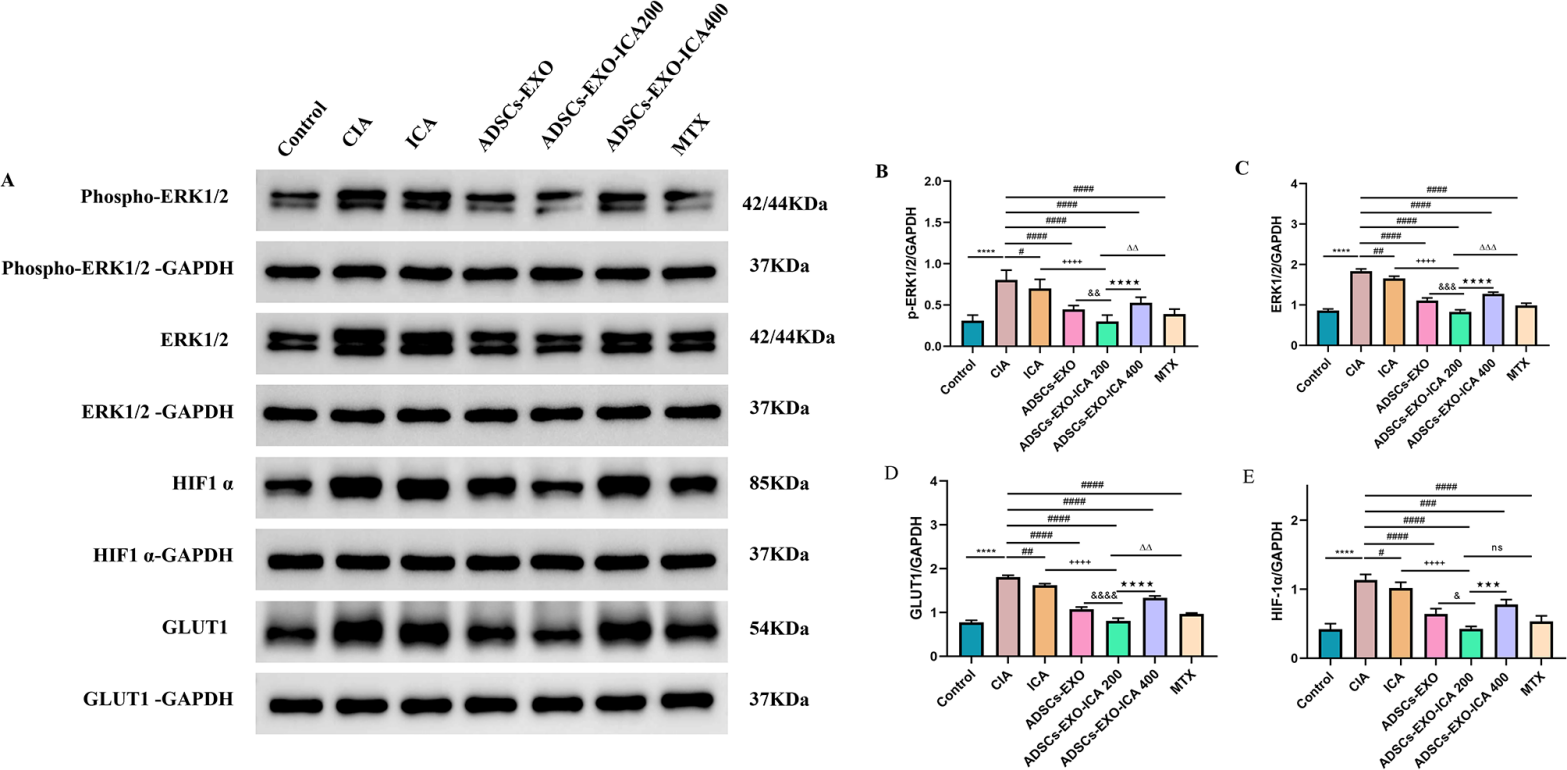
ADSCs-EXO-ICA200 reduces macrophage polarization towards the M1-type by inhibiting the ERK1/2/HIF-1α/GLUT1 pathway-mediated macrophage glycolysis. (**A**) Western blot (WB) images showing the expression of GLUT1, HIF-1α, ERK1/2, and p-ERK1/2 proteins. (**B-I**) Relative protein expression levels of GLUT1, HIF-1α, ERK1/2, and p-ERK1/2. Data were expressed as mean ± SD (*n* = 3, ^****^p < 0.0001 vs. the Control group, ^#^*p* < 0.05, ^##^*p* < 0.01, ^###^*p* < 0.001 and ^####^*p* < 0.0001 vs. the CIA group, ^&^*p* < 0.05, ^&&^*p* < 0.01, ^&&&^*p* < 0.001,and ^&&&&^*p* < 0.0001 vs. the ADSCs-EXO group, ^++++^*p* < 0.0001 vs. the ICA group, ••• *p* < 0.001 and •••• *p* < 0.0001 vs. the ADSCs-EXO-ICA200 group, ^ΔΔ^*p* < 0.01 and ^ΔΔΔ^*p* < 0.001 vs. the ADSCs-EXO-ICA200 group, ns = no significance)

To further explore the relationship between glycolysis in M1-type macrophages and the ERK1/2/HIF-1α/GLUT1 signaling pathway (Fig. 10A-E), the protein expression of GLUT1, HIF-1α, ERK1/2, and p-ERK1/2 was analyzed by Western Blot (WB). In the CIA group, the levels of GLUT1, HIF-1α, ERK1/2, and p-ERK1/2 were significantly higher compared to the control group. However, treatments with ICA, ADSCs-EXO, ADSCs-EXO-ICA200, ADSCs-EXO-ICA400, and MTX markedly reduced the protein expression of these components, with ADSCs-EXO-ICA200 showing the most pronounced effect. These results suggest that ADSCs-EXO-ICA200 reduces glycolysis in M1-type macrophages by inhibiting the ERK1/2/HIF-1α/GLUT1 pathway.

In summary, ADSCs-EXO-ICA200 effectively modulates the M1-to-M2 phenotypic switch by down-regulating glycolysis levels through blocking the ERK/HIF-1α/GLUT1 pathway in vivo.

### Safety evaluation of ADSCs-EXO-ICA

**Fig. 11:**
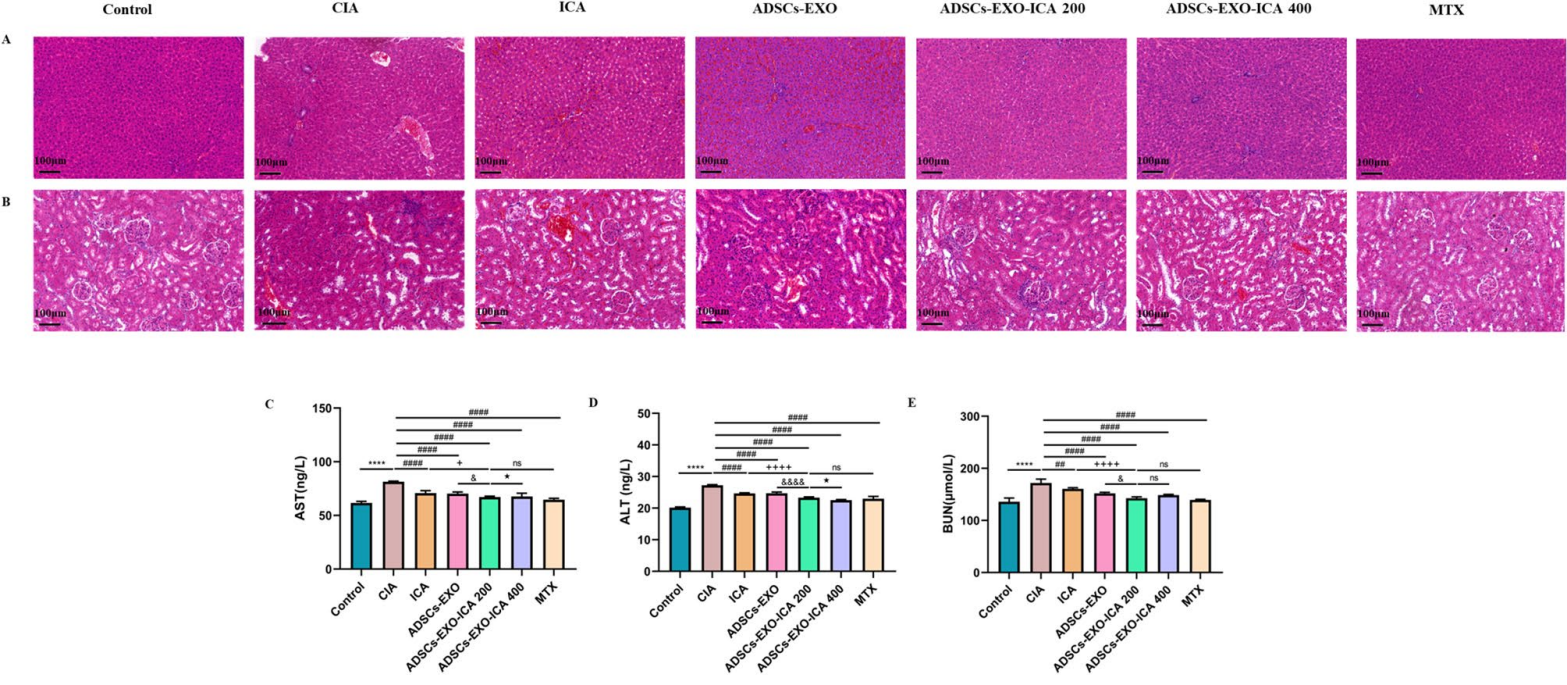
(**A-B)** H&E staining of liver and kidney across different groups (scale bar = 100 μm). (**C-E**) Biochemical indicators of liver and kidney function across different groups. Data were expressed as mean ± SD (*n* = 3, ^****^p < 0.0001 vs. the Control group, ^##^*p* < 0.01 and ^####^*p* < 0.0001 vs. the CIA group, ^&^*p* < 0.05 and ^&&&&^*p* < 0.0001 vs. the ADSCs-EXO group, ^+^*p* < 0.05 and ^++++^*p* < 0.0001 vs. the ICA group, •*p* < 0.05 vs. the ADSCs-EXO-ICA200 group, ns = no significance)

To assess the safety of ADSCs-EXO-ICA, we evaluated both pathological changes and functional alterations in the liver and kidneys. Pathological changes were assessed using hematoxylin and eosin (HE) staining, while functional changes, including levels of alanine aminotransferase (ALT), aspartate aminotransferase (AST), and blood urea nitrogen (BUN), were measured using ELISA. One week after the second immunization, liver (ALT and AST) and kidney (BUN) functions in the CIA group were significantly elevated compared to the normal group (Fig. 11C-E). However, these biological indicators were significantly mitigated by treatments with ICA, ADSCs-EXO, ADSCs-EXO-ICA200, ADSCs-EXO-ICA400, and MTX, particularly with ADSCs-EXO-ICA200. No obvious pathological changes were observed in the liver or kidneys (Fig. 11A-B). These results indicate that ADSCs-EXO-ICA200 can effectively prevent functional damage to the liver and kidneys, demonstrating its safety.

## Discussion

The objective of this study was to investigate a novel drug delivery system, ADSCs-EXO-ICA, which co-delivers drugs to facilitate the M1-to-M2 phenotypic switch by down-regulating glycolysis through blocking the ERK/HIF-1α/GLUT1 pathway, thus mitigating the RA progression. During the pathogenesis of synovitis in RA, M1-type macrophages are recruited to the arthritic area, triggering a cascade of inflammatory factors that exacerbate the condition [39, 40]. Numerous studies have established that macrophage metabolic pathways are intricately linked to their phenotype and function [41, 42]. Predominantly, M1-type macrophages rely on glycolysis, which not only perpetuates the release of inflammatory factors but also promotes their polarization toward the M1 phenotype [43]. Although many current anti-rheumatic drugs target macrophage glycolysis to reduce RA severity [44], their side effects and adverse reactions cannot be overlooked [45]. Furthermore, research has shown that the proportion of M1-type macrophages exceeds that of M2-type both in RA patients and in the CIA model [46, 47]. Consequently, we developed a promising, nanoscale, and safe drug delivery system aimed at evaluating its efficacy in modulating M1-M2 polarization in RA.

We have successfully developed a drug delivery system, ADSCs-EXO-ICA. Initially, ADSCs-EXO were isolated via ultracentrifugation and characterized biologically using TEM, NTA, and WB. The results confirmed that ADSCs-EXO displayed the typical morphological and molecular characteristics of exosomes [48]. Subsequently, we combined ADSCs-EXO with icariin (ICA) to create ADSCs-EXO-ICA and assessed its encapsulation efficiency. The encapsulation rate of ADSCs-EXO-ICA reached as high as 92.4 ± 0.008%%, significantly surpassing that of BMSC-EXO loaded with ICA, which achieved up to 82%, thus demonstrating that ADSCs-EXO significantly enhances the drug-loading capacity of ICA. Furthermore, the cellular uptake rate of ADSCs-EXO-ICA by M1-type macrophages was higher than that of ADSCs-EXO alone, indicating that ADSCs-EXO-ICA improves the cellular uptake efficiency of M1-type macrophages. These experimental results suggest that ADSCs-EXO-ICA was successfully engineered and possesses a high encapsulation rate, which is foundational for the phagocytosis of ADSCs-EXO-ICA by M1-type macrophages, facilitating its pharmacological effects. ADSCs-EXO-ICA exhibited potent anti-inflammatory effects and the ability to modulate macrophage polarization, likely due to the synergistic benefits of its drug delivery system. Additionally, combining therapy with resveratrol and celastrol with exosomes has been shown to enhance efficacy in treating sepsis [49].

To further validate the pharmacological efficacy of ADSCs-EXO-ICA in vivo, the classical RA model, CIA, was employed [50]. We observed the biodistribution of DiR@ADSCs-EXO, DiR@ADSCs-EXO-ICA200, and DiR@ADSCs-EXO-ICA400 in CIA rat. All three groups predominantly accumulated in the liver and spleen, consistent with previous findings [51, 52], which might result from the liver and spleen being sensitive to blood vessels and containing multiple types of tissue macrophages, but a notably high quantity of DIR@ADSCs-EXO-ICA200 was detected in the damaged arthritic areas compared to DiR@ADSCs-EXO. Additionally, ADSCs-EXO-ICA200 demonstrated greater accumulation at the ankle joint than the other two groups, establishing a foundation for its pharmacological efficacy in synovitis.

ADSCs-EXO-ICA showed the strongest anti-inflammatory effects in early arthritis and significantly influenced macrophage polarization in CIA rats. The anti-inflammatory efficacy of ADSCs-EXO-ICA200 was preliminarily found to be superior to that of single administration, better than ADSCs-EXO-ICA400, and comparable to MTX, consistent with in vitro results. This phenomenon tentatively suggests that ADSCs-EXO-ICA200 achieved the most effective anti-inflammatory impact, benefiting from the synergistic effects of ADSCs-EXO and ICA. Additionally, ADSCs-EXO-ICA200 demonstrated robust effects on adjusting macrophage polarization in CIA rats, aligning with in vitro findings. Interestingly, ADSCs-EXO-ICA200 was more potent than ADSCs-EXO-ICA400, was more concentrated in the liver in ADSCs-EXO-ICA400 and less enriched in the ankle joint. Likely, because ICA, being poorly water-soluble and fat-soluble with slow metabolism. After liposome-carrying ICA altered pharmacokinetics and enhanced distribution of ICA in different tissues [53]. And mounting research had demonstrated the ameliorative effects of ICA on liver diseases probably related precisely to the accumulation of ICA and the metabolism of the flavonoids in the liver [54–56]. It was also possible that because the ADSCs-EXO-ICA400 needed to take a longer time to reside after being cleared by liver-resident macrophages and hepatic sinusoidal endothelial cells than the ADSCs-EXO-ICA200, and therefore a smaller amount accumulates in the joints in the same time [57].

It is well known that the tissue damage caused by RA is irreversible, and the continued progression of synovial inflammation leads to cartilage erosion and bone destruction [58]. In this study, ADSCs-EXO-ICA was able to partially inhibit existing bone damage. Thus, we demonstrated that ADSCs-EXO-ICA has the potential to inhibit the glycolytic process of macrophages in the joints and suppress macrophage polarization toward the M1-type, which in turn helps prevent bone destruction in CIA rats.

To investigate the mechanism of ADSCs-EXO-ICA, we conducted cellular gene transcriptomics to identify potential targets. KEGG pathway analysis indicated that molecules such as MAPK, HIF-1α, and glycolysis are potential targets through which ADSCs-EXO-ICA may exert anti-inflammatory effects, aligning with previous research findings. Interestingly, we observed a reduction in ERK activity following ADSCs-EXO-ICA treatment, which appeared to decrease HIF-1α levels. Prior studies have highlighted ERK as a valuable target for RA treatment [59–61], and the therapeutic effects of ERK knockdown were similar to those observed with ADSCs-EXO-ICA. Additionally, one study reported that an ERK inhibitor suppressed M1 macrophages in RA [62, 63]. ERK is known to influence macrophage polarization and metabolic reprogramming [64], and plays a crucial role in the RAS/RAF/ERK cascade of the MAPK signaling pathway. Our KEGG results led us to hypothesize that the regulatory effect of ADSCs-EXO-ICA on M1-type macrophages might be related to ERK targets within the MAPK signaling pathway. Furthermore, the HIF-1α signaling pathway is central to macrophage glycolysis and M1-type polarization in RA [65, 66], consistent with our findings. Upregulation of HIF-1α not only enhances the transcription of genes involved in the macrophage glycolysis pathway (e.g., GLUT1) but also regulates enzymes essential for macrophage glycolysis (e.g., LDHA, HKII, and PKM2) [67]. Therefore, we speculate that the ERK1/2/HIF-1α/GLUT1 signaling pathway may underlie the mechanism by which ADSCs-EXO-ICA modulates macrophage polarization and suppresses macrophage glycolysis. PCR results confirmed that ADSCs-EXO-ICA could facilitate the M1-to-M2 phenotypic switch by down-regulating glycolysis through inhibiting the ERK/HIF-1α/GLUT1 pathway, thereby alleviating inflammation in vitro.

We had firstly developed the drug delivery system-ADSCs-EXO-ICA, which had not been clinically studied. Obviously, the anti-inflammatory effect of ADSCs-EXO-ICA was better than that of ADSCs-EXO, and it was very potential to be applied in clinical studies. However, the limitations of applying this drug delivery system were the low availability of ADSCs-EXO and the unknown safe dose in humans. Therefore, we need to explore new methods to increase the yield of ADSCs-EXO and multi-center clinical studies with large samples are needed to explore the effective dose in future.

In conclusion, ADSCs-EXO-ICA can adjust the M1-to-M2 phenotypic switch to alleviate RA by inhibiting glycolysis through blocking the ERK/HIF-1α/GLUT1 pathway. ADSCs-EXO-ICA provides an effective therapeutic strategy for treating synovial inflammation and bone destruction in RA by modulating metabolic pathways to reverse cell type in the treatment of RA.

## Materials and methods

### Cell and animals

RAW264.7 macrophages were acquired from Shanghai Fuheng Biotechnology Co. These cells were cultured in Dulbecco’s Modified Eagle Medium (DMEM) supplemented with 10% fetal bovine serum (FBS) and maintained at 37℃ in a 5% CO_2_ atmosphere. To induce M1-type macrophage polarization, LPS (100 ng/mL) and IFN-γ (20 ng/mL) were added to the culture for 24 h. Third-generation ADSCs were cultured in MSC-specific medium devoid of exosome fetal bovine serum (FBS). Upon reaching 80–90% confluence in T75 flasks, the supernatant was collected. The use of primary human adipose stem cells (ADSCs) were obtained from Peking Union Medical College, had been approved by the local ethical committee of the Institute of the Academic Committee of the Chinese Academy of Medical Sciences and Peking Union Medical College Hospital and informed written consent was obtained from the patient [68]. Male SD rats, aged 6–8 weeks, were obtained from Beijing Vital River Laboratory Animal Technology Co., Ltd., and housed in a specific pathogen-free (SPF) environment under a 12-hour light/dark cycle. Both food and water were provided ad libitum. All animal care and experimental procedures adhered to the guidelines approved by the Ethics Committee of the Institute of Basic Theory for Chinese Medicine at the China Academy of Chinese Medical Sciences (Approval No. IBTCMCACMS2- 2209-05).

### Cell grouping

To evaluate the efficacy of different treatments on M1-type macrophages, the experiment was organized into several groups: the Blank group, the LPS + IFN-γ group, the ICA group (40 µg/mL), the ADSCs-EXO group (40 µg/mL), and the ADSCs-EXO-ICA group (80 µg/mL).

### Animal grouping

The rats in the modeling group were randomly assigned into six groups: Blank received saline(100µL), CIA, ICA (100µL, 1 µg/µL), ADSCs-EXO [69] (100µL, 1 µg/µL), ADSCs-EXO-ICA200 (200µL, 1 µg/µL), ADSCs-EXO-ICA400 (200µL, 2 µg/µL), and methotrexate (MTX, 100µL, 1 mg/kg) [70], with 10 rats in each group. All groups of rats were injected via tail vein every other day for a total of eight injections.

### Synthesis and characterization of ADSCs-EXO-ICA

Extraction of ADSCs-EXO: Exosomes were isolated using the ultracentrifugation method. Specifically, the supernatant underwent sequential centrifugation at 300×g at 4℃ for 10 min, 2000×g for 20 min, and 12,000×g for 40 min, 100,000 g for 70 min, and 150,000 g two times for 80 min [71]. After centrifugation, the supernatant was discarded, and 300μl of PBS was added to resuspend the ADSCs-EXO and, which were then stored at -80 °C.

Synthesis of ADSCs-EXO-ICA: ICA dissolved with dimethyl sulfoxide (DMSO ≤ 1‰). Equal quantities of ADSCs-EXO and ICA were co-incubated for 2 h at room temperature and kept the final solvent concentration ≤ 10%. Mixed ICA solution with exosome dispersion, underwent centrifugation at 1,0000 × g for 10 min to remove the free drug, followed by centrifugation at 13,500 g for 2 min, discarded the supernatant, resuspended in 100μL PBS for three times and filtered through a 0.22 μm filter to obtain drug-loaded exosomes (ADSCs-EXO-ICA) [72–76].

Characterization of ADSCs-EXO and ADSCs-EXO-ICA: TEM was employed to assess the morphology of both ADSCs-EXO and ADSCs-EXO-ICA. Nanoparticle tracking analysis (NTA) was used to determine the particle size of the exosomes. Western blot (WB) analysis was utilized to detect exosome signature proteins CD9, CD63 and TSG101.

### Encapsulation rate of ADSCs-EXO-ICA

A detection method was established using the following chromatographic conditions: a C18 column (4.6 mm × 250 mm, 5 μm); column temperature: 25 °C; mobile phase: acetonitrile: water at a ratio of 95:5; flow rate: 1.2 mL/min; detection wavelength: 270 nm; injection volume: 5 µl. ICA was monitored from 200 to 400 nm using PDA-UV. The standard curve of ICA was utilized to calculate the content of ICA loaded in ADSCs-EXO, thereby determining its loading efficiency. Data were analyzed using Origin software.

### Cellular uptake of ADSCs-EXO-ICA

RAW264.7 cells were seeded in culture dishes at a density of 1 × 10^6^ cells per well, with or without LPS and IFN-γ, and incubated at 37 °C for 24 h. ADSCs-EXO and ADSCs-EXO-ICA were labeled following the instructions of the PKH67 dye kit. PKH67-labeled exosomes were isolated by ultracentrifugation at 100,000 g for 70 min and then co-cultured with macrophages for 8 h. Subsequently, cells were fixed, stained with DAPI, and imaged using a laser scanning confocal microscope (Olympus, Japan).

### Cell immunofluorescence

RAW264.7 cells were seeded onto coverslips in 12-well plates (5.0 × 10^4^ cells/well) overnight, stimulated with LPS and IFN-γ for 24 h, then subsequently treated with ICA (40 µg/mL), ADSCs-EXO (40 µg/mL), and ADSCs-EXO-ICA (80 µg/mL) groups for 48 h. Cells were incubated overnight with anti-inducible nitric oxide synthase (iNOS, 1:200, Proteintech) antibody, followed by a FITC-conjugated secondary antibody (1:2000). Cells were then fixed, stained with DAPI, and imaged using a laser scanning confocal microscope. Images were analyzed with ImageJ software.

### Flow cytometry

RAW264.7 cells (1.0 × 10^6^ cells/dish) were plated in culture dishes overnight, induced with LPS and IFN-γ for 24 h, and subsequently the cells were treated with various formulations for 48 h. Cells were then incubated with FITC-conjugated anti-F4/80 antibody (1:200, eBioscience, Thermo Fisher Scientific, Waltham, MA) and PE-conjugated anti-CD86 antibody (1:50, eBioscience) at 4 °C for 30 min, followed by two washes. Detection was performed using a CytoFLEX flow cytometer (Beckman Coulter, USA), and data were analyzed using FlowJo software. Experiments were conducted at least three times.

### Enzyme-linked immunosorbent assay (ELISA)

RAW264.7 cells were seeded into the culture dishes. After incubation with LPS (100 ng/mL) and IFN-γ (20 ng/mL) for 24 h, the cells were treated with various formulations for 48 h. Subsequently, cell supernatants were collected, and the concentrations of cytokines (TNF-α, IL-6, and IL-10) and glycolysis-related indicators (LA, SA, CA, G6PD, and HKII) were quantified using ELISA kits following the manufacturer’s instructions. For in vivo analysis, levels of CRP, RF, CII, TNF-α, IL-1β, IL-6, and IL-10 in serum were assessed at the end of the experimental treatment.

### Transcriptome sequencing analysis

Freshly sorted macrophages (> 5 × 10^6^ cells) were lysed using Trizol reagent (Takara) and then transported on dry ice to Allwegene for sequencing. A total of five sets of macrophage samples were sequenced. Gene expression levels for each sample were analyzed using HTSeq (v0.5.4 p3) software with the model set to union. GO enrichment analysis was performed using GOseq (v1.22). KEGG analysis identified key biochemical metabolic pathways and signaling pathways involved in differentially expressed genes via Pathway significance enrichment. For samples with biological replicates, differential expression analysis was conducted using DESeq (v1.10.1) (Anders et al., 2010). The P-values from the differential expression analysis were adjusted for false discovery rate (FDR) using the Benjamini and Hochberg method. The criteria for differential gene screening were generally: p-value < 0.05.

### Western blotting (WB)

Western blotting was used to detect the expression of iNOS, LDHA, PKM2, HKII GLUT1, HIF-1α, ERK1/2, and p-ERK1/2. Cells treated with different formulations were washed with precooled PBS, lysed with 100ul of RIPA buffer (containing 1% PMSF + 1% phosphatase inhibitor), and centrifuged at 15000r for 15 min. A total of 20 µg of protein, assessed using a BCA Protein Assay Kit, was separated by electrophoresis on a 10% SDS-PAGE gel at 80 mV for 30 min, followed by 120 mV for 1 h, and then transferred to PVDF membranes at 300 mA for 120 min. The membranes were subsequently blocked with 5% nonfat dry milk for 1 h and incubated overnight at 4 °C with primary antibodies against iNOS (1:2000, Proteintech, China), Arg1 (1:10,000, Proteintech, China), LDHA (1:15,000, Proteintech), PKM2 (1:2000, Proteintech), HKII (1:25,000, Proteintech), GLUT1 (1:5000, Abcam), HIF-1α (1:5000, Abcam), ERK1/2 (1:4500, Proteintech), and p-ERK1/2 (1:1000, Abcam). The next day, membranes were incubated with goat anti-rabbit (1:3000, Proteintech) and goat anti-mouse antibodies (1:3000, Proteintech) at room temperature for 1 h. The protein expressions of iNOS, LDHA, PKM2, HKII, GLUT1, HIF-1α, ERK1/2, and p-ERK1/2 were detected using the Bio-Rad ChemiDoc XRS System, with GAPDH serving as an endogenous control.

### Quantitative reverse transcription-polymerase chain reaction (RT-qPCR)

Macrophages from the aforementioned five groups were collected to assess the expression of M1-type macrophage and ERK1/2/HIF-1α/GLUT11 pathway-related genes. Total RNA was extracted following standard procedures and the concentration was measured. The RNA was then reverse transcribed into cDNA using a reverse transcription kit (Takara). The qPCR reaction was prepared using the SYBR Green Master Mix kit (Takara) according to the manufacturer’s instructions. Glyceraldehyde-3-phosphate dehydrogenase (GAPDH) was used as the internal reference. Gene expression quantification was performed using the 2^−ΔΔ Ct^ method. The primer sequences for each gene are detailed in Table 1 below.

Table 1 Reverse transcription-polymerase chain reaction primer sequences.

**Table 1:** Primer sequences

Target gene	F primer sequences (5′-3′)	*R* primer sequence (5′-3′)
GAPDH	AGGTCGGTGTGAACGGATTTG	GGGGTCGTTGATGGCAACA
TNF-α	CTGAACTTCGGGGTGATCGG	GGCTTGTCACTCGAATTTTGAG
TGF-β	GGCTTGTCACTCGAATTTTGAGA	GCCTTAGTTTGGACAGGATCTG
p-ERK	CAGGTGTTCGACGTAGGGC	TCTGGTGCTCAAAAGGACTGA
ERK	GCGGCTACGACGAGAACAT	GGCTAAGTCAAAATCAGCCTCA
G6PD	CACAGTGGACGACATCCGAAA	AGCTACATAGGAATTACGGGCAA
HK2	TGATCGCCTGCTTATTCACGG	AACCGCCTAGAAATCTCCAGA
HIF-1α	ACCTTCATCGGAAACTCCAAAG	CTGTTAGGCTGGGAAAAGTTAGG
LDHA	TGTCTCCAGCAAAGACTACTGT	GACTGTACTTGACAATGTTGGGA
GLUT-1	CAGTTCGGCTATAACACTGGTG	GCCCCCGACAGAGAAGATG

### Establishment of collagen-induced arthritis (CIA) rat models

Sixty male SD rats of SPF grade, aged 2 months, were used. Equal volumes of bovine type II collagen solution (2 mg/mL) and incomplete Freund’s adjuvant (IFA) were mixed in an ice bath to form an emulsion at a final concentration of 1 mg/mL. For initial immunization, 100 µL of this emulsion was injected intradermally at the base of the tail. One week after the initial immunization, a booster immunization was administered in the same manner. Two weeks after the initial immunization, a CIA model in rats was successfully established.

### Biodistribution in CIA rats

Purified ADSCs-EXO and ADSCs-EXO-ICA in PBS were incubated with 1 mM DiR (MCE, USA) (which is a fluorescent lipophilic tracer) for 30 min at room temperature. The labeled ADSCs-EXO and ADSCs-EXO-ICA were then separated from unbound DiR by ultracentrifugation at 100,000 g for 2 h at 4℃ [77, 78]. DiR-labeled exosomes (DiR@ADSCs-EXO, DiR@ADSCs-EXO-ICA200, ADSCs-EXO-ICA400) were administered via the tail veins of CIA rats. The biodistribution of DiR@ADSCs-EXO (100µL,1 µg/µL), DiR@ADSCs-EXO-ICA200 (200µL,1 µg/µL), ADSCs-EXO-ICA400 (200µL,2 µg/µL) in the ankle joints was assessed through in vivo imaging of fluorescence intensity using the Carestream MI System (Carestream Health Inc., USA) at specified time points (1, 2, 4, 6, 8, 12, and 24 h). After imaging, the rats were sacrificed, and their hearts, livers, spleens, lungs, kidneys, and paws were collected for further in vivo imaging. Fluorescence intensity was quantified using Image J (Carestream Health Inc., USA). Rats treated with DiR@ADSCs-EXO served as controls.

### Immunofluorescence staining

Ankle joints from CIA rats were collected 24 h after the final administration to prepare sections. Sections of 10 μm thickness were stained with iNOS antibody (1:2000, proteintech). Nuclei was stained by DAPI. A laser scanning confocal microscope (NIKON Eclipse ci, Japan) was used to record the fluorescent distributions in the ankle joints.

### Micro-computed tomography (Micro-CT) imaging

All rats were sacrificed on day 28 after arthritis induction and then fixed in 4% paraformaldehyde. The knee and ankle joints were scanned using a micro-CT scanner at a voltage of 70 kV and a current of 200 µA, with a scanning resolution of 10.2 μm. The knee joint was scanned from the lowest point of the lateral growth plate of the tibial knee as the baseline, and a 3 mm thick area of the bone marrow cavity below the baseline was designated as the region of interest (ROI) for 3D reconstruction. For the ankle scans, 1451 consecutive slices covering a 14.8 mm thickness from the heel bone, i.e., the tarsal region, were set as the ROI for 3D reconstruction. 3D images were reconstructed using NRecon software and analyzed using various analysis software. Quantitative analyses were performed to assess morphometric characteristics such as bone mineral density (BMD), bone surface to volume ratio (BS/BV), trabecular separation (Tb.Sp), and trabecular bone thickness (Tb.Th).

### Histopathological examination

Twenty-eight days after arthritis induction, all rats were sacrificed. Ankle joints were decalcified in 15% tetrasodium ethylenediaminetetraacetic acid for 2 months, then processed for paraffin embedding and sectioned at 3 μm thickness. Sections were stained using Hematoxylin-eosin (HE) staining [79], Safranin O-Fast Green staining [80], TRAP staining methods [81]. A light microscope (Leica Biosystems, Germany) was utilized for observation. Additionally, joint tissues were assessed and scored for cellular infiltration, bone erosion, and synovial hyperplasia on a scale of 0–4. Scores were evaluated and the mean grade was calculated.

### Safety evaluation

Healthy rats (200 ± 20 g) were intravenously injected with treatments including Blank, CIA, ICA (100 µg per rat), ADSCs-EXO (100 µg per rat), ADSCs-EXO-ICA200 (200 µg per rat), ADSCs-EXO-ICA400 (400 µg per rat), or methotrexate (2 mg/mL MTX) to assess the safety of ADSCs-EXO-ICA. After the final dose, the rats were euthanized, and key organs such as the liver and kidneys were collected for histological analysis as previously described. Levels of alanine aminotransferase (ALT), aspartate aminotransferase (AST), blood urea nitrogen (BUN) [82] in the serum were measured using a Labsystems Multiskan MS.

### Statistical evaluation

Data were analyzed using SPSS 25.0 software (Statistical Package for the Social Sciences, USA). Graphs were created with GraphPad Prism 8 software. The quantitative results were provided as mean ± standard. Comparisons between two groups were conducted using paired t-tests, while comparisons among multiple groups were performed using one-way ANOVA. Statistical differences were considered significant at *p* < 0.05.

## Data Availability

The datasets used and/or analyzed during the current study are available from the corresponding author upon reasonable request.

## References

[1] Smolen JS, Aletaha D, McInnes IB (2016). Lancet.

[2] Boutet MA, Courties G, Nerviani A, Le Goff B, Apparailly F, Pitzalis C. F. Blanchard Autoimmun Rev 2021,20,102758. 10.1016/j.autrev.2021.102758 33476818

[3] Komatsu N (2022). H Takayanagi Nat Rev Rheumatol.

[4] Tu J, Huang W, Zhang W, Mei J, Zhu C (2021). Front Immunol.

[5] Takeuchi T, Yoshida H (2021). S Tanaka Autoimmun Rev.

[6] Udalova IA, Mantovani A. M. Feldmann Nat Rev Rheumatol 2016,12,472 – 85. 10.1038/nrrheum.2016.91 27383913

[7] Burmester GR (2017). J E Pope Lancet.

[8] Onuora S (2016). Nat Rev Rheumatol.

[9] Liu X, Wang Z, Qian H, Tao W, Zhang Y, Hu C, Mao W. Q Guo Front Immunol 2022,13945129. 10.3389/fimmu.2022.945129 PMC9376257 35979373

[10] Zhang J, Fan F, Liu A, Zhang C, Li Q, Zhang C, He F, Shang M (2022). Front Pharmacol.

[11] Luo Z, Dong J (2022). J Wu Int Immunopharmacol.

[12] Bi Z, Zhang W. X. Yan Biomed Pharmacother 2022,151113180. 10.1016/j.biopha.2022.113180 35676785

[13] Zeng J, Sun P, Zhao Y, Fang X, Wu Z (2023). X Qi Asian J Pharm Sci.

[14] Yu X, Dong M, Wang L, Yang Q, Wang L, Han W, Dong J, Liu T, Kong Y, Niu W (2023). Drug Deliv.

[15] Liu J, Cheng Q, Wu X, Zhu H, Deng X, Wang M, Yang S, Xu J, Chen Q, Li M, Liu X. C. Wang Cells 2022,11,. 10.3390/cells11244091 PMC9777100 36552853

[16] Chen M, Lu L, Cheng D, Zhang J, Liu X, Zhang J. T. Zhang Molecules 2023,28,. 10.3390/molecules28135128 PMC10343815 37446790

[17] Desai TD, Wen YT, Daddam JR, Cheng F, Chen CC, Pan CL, Lin KL. R. K. Tsai Bioeng Transl Med 2022,7,e10289. 10.1002/btm2.10289 PMC9115698 35600664

[18] Guangtao F, Zhenkang W, Zhantao D, Mengyuan L, Qingtian L, Yuanchen M, Yuanfeng C, Qiujian Z (2021). Front Pharmacol.

[19] Cai Y, Li J, Jia C, He Y (2020). C Deng Stem Cell Res Ther.

[20] Harasymiak-Krzyżanowska I, Niedojadło A, Karwat J, Kotuła L, Gil-Kulik P, Sawiuk M, Kocki J (2013). Cell Mol Biol Lett.

[21] Qin Y, Ge G, Yang P, Wang L, Qiao Y, Pan G, Yang H, Bai J, Cui W, Geng D (2023). Adv Sci (Weinh).

[22] Tan F, Li X, Wang Z, Li J, Shahzad K. J Zheng Signal Transduct Target Ther 2024,917. 10.1038/s41392-023-01704-0 PMC10784577 38212307

[23] Choi EW, Seo MK, Woo EY, Kim SH, Park EJ (2018). S Kim Exp Dermatol.

[24] Fang Y, Ni J, Wang YS, Zhao Y, Jiang LQ, Chen C, Zhang RD, Fang X, Wang P, Pan H. F. Autoimmun Rev. 2023;22:103260. 10.1016/j.autrev.2022.103260 36565798

[25] Tavasolian F, Moghaddam AS, Rohani F, Abdollahi E, Janzamin E, Momtazi-Borojeni AA, Moallem SA, Jamialahmadi T, Sahebkar A (2020). Autoimmun Rev.

[26] Chang TH, Wu CS, Chiou SH, Chang CH. H. J. Liao Biomedicines 2022,10. 10.3390/biomedicines10071725 PMC9312519 35885030

[27] Liu Y, Xu R, Gu H, Zhang E, Qu J, Cao W, Huang X, Yan H (2021). J He Z Cai Biomark Res.

[28] De Jesus A, Keyhani-Nejad F, Pusec CM, Goodman L, Geier JA, Stoolman JS, Stanczyk PJ, Nguyen T, Xu K, Suresh KV, Chen Y, Rodriguez AE, Shapiro JS, Chang HC, Chen C, Shah KP, Ben-Sahra I, Layden BT, Chandel NS, Weinberg SE (2022). H Ardehali Mol Cell.

[29] Umar S, Palasiewicz K, Volin MV, Romay B, Rahat R, Tetali C, Arami S, Guma M, Ascoli C, Sweiss N, Zomorrodi RK, O’Neill LAJ (2021). S Shahrara Cell Mol Life Sci.

[30] Li Y, Li YC, Liu XT, Zhang L, Chen YH, Zhao Q, Gao W, Liu B, Yang H (2022). P Li Cell Rep.

[31] Kim H, Back JH, Han G, Lee SJ, Park YE, Gu MB, Yang Y, Lee JE. S. H. Kim Biomaterials 2022,286,121578. 10.1016/j.biomaterials.2022.121578 35594838

[32] Choudhry H (2018). L Harris Cell Metab.

[33] Mills CN, Joshi SS (2009). R M Niles Mol Cancer.

[34] Meng M, Wang L, Yao Y, Lin D, Wang C, Yao J, Sun H. M. Liu Phytomedicine 2023,119,155010. 10.1016/j.phymed.2023.155010 37586160

[35] Arseni C, Samiotaki M, Panayotou G, Simos G (2024). I Mylonis Cell Mol Life Sci.

[36] Macintyre AN, Gerriets VA, Nichols AG, Michalek RD, Rudolph MC, Deoliveira D, Anderson SM, Abel ED, Chen BJ, Hale LP (2014). J C Rathmell Cell Metab.

[37] Garcia-Carbonell R, Divakaruni AS, Lodi A, Vicente-Suarez I, Saha A, Cheroutre H, Boss GR, Tiziani S, Murphy AN. M. Guma Arthritis Rheumatol 2016,68,1614–26. 10.1002/art.39608 PMC4963240 26815411

[38] Zhou L, Wang Y, Zhou M, Zhang Y, Wang P, Li X, Yang J, Wang H. Z. Ding Nat Commun 2018,9,1480. 10.1038/s41467-018-03914-5 PMC5902613 29662084

[39] Yang Y, Guo L, Wang Z, Liu P, Liu X. J Ding W Zhou Biomaterials 2021,264120390. 10.1016/j.biomaterials.2020.120390 32980634

[40] Zhang L, Meng W, Chen X, Wu L, Chen M, Zhou Z, Chen Y, Yuan L, Chen M, Chen J, Shui P (2023). ACS Appl Mater Interfaces.

[41] Kratz M, Coats BR, Hisert KB, Hagman D, Mutskov V, Peris E, Schoenfelt KQ, Kuzma JN, Larson I, Billing PS, Landerholm RW, Crouthamel M, Gozal D, Hwang S. P. K. Singh,L. Becker Cell Metab 2014,20,614 – 25. 10.1016/j.cmet.2014.08.010 PMC4192131 25242226

[42] Na YR, Je S (2018). S H Seok Cancer Lett.

[43] Soto-Heredero G, Gómez de Las Heras MM, Gabandé-Rodríguez E (2020). J Oller M Mittelbrunn Febs j.

[44] Hu F, Shi L, Liu X, Chen Y, Zhang X, Jia Y, Liu X, Guo J, Zhu H, Liu H, Xu L, Li Y, Wang P, Fang X, Xue J, Xie Y, Wei C, Song J, Zheng X, Liu YY, Li Y, Ren L, Xu D, Lu L, Qiu X, Mu R, He J, Wang M, Zhang X, Liu W. Z. Li Ann Rheum Dis 2024,. 10.1136/ard-2023-224878 38302261

[45] Rubbert-Roth A, Szabó MZ, Kedves M, Nagy G, Atzeni F. P. Sarzi-Puttini Autoimmun Rev 2019,18102398. 10.1016/j.autrev.2019.102398 31639514

[46] Zerrouk N, Alcraft R, Hall BA, Augé F. Niarakis NPJ Syst Biol Appl 2024,1010. 10.1038/s41540-024-00337-5 PMC10811231 38272919

[47] Tan T, Huang Q, Chu W, Li B, Wu J, Xia Q (2022). X Cao Drug Deliv.

[48] Kalluri R. V. S. LeBleu Sci 2020,367. 10.1126/science.aau6977 PMC7717626 32029601

[49] Zheng X, Xing Y, Sun K, Jin H, Zhao W, Yu F. Adv Healthc Mater 2023,12e2301325. 10.1002/adhm.202301325 37530416

[50] Luross JA. N. A. Williams Immunology 2001,103,407 – 16. 10.1046/j.1365-2567.2001.01267.x PMC1783255 11529930

[51] Faruqu FN, Wang JT, Xu L, McNickle L, Chong EM, Walters A, Gurney M, Clayton A, Smyth LA, Hider R. J. Sosabowski,K. T. Al-Jamal Theranostics 2019,9,1666–82. 10.7150/thno.27891 PMC6485196 31037130

[52] Morishima Y, Kawabori M, Yamazaki K, Takamiya S, Yamaguchi S, Nakahara Y, Senjo H, Hashimoto D, Masuda S, Fujioka Y, Ohba Y, Mizuno Y, Kuge Y. M Fujimura Int J Mol Sci 2024,25. 10.3390/ijms25042406 PMC10889446 38397083

[53] Yang W, Yu XC, Chen XY, Zhang L, Lu CT, Zhao (2012). J Pharm Pharmacol.

[54] Zhao H, Tian H. J Biochem Mol Toxicol 2024,38e23566. 10.1002/jbt.23566 37888945

[55] Ma Y, Zhao C, Hu H. S. Yin Phytochemistry 2023,215,113841. 10.1016/j.phytochem.2023.113841 37660725

[56] Algandaby MM, Breikaa RM, Eid BG, Neamatallah TA, Abdel-Naim AB. O. M. Ashour Pharmacol Rep 2017,69,616–24. 10.1016/j.pharep.2017.02.016 28505603

[57] Kim J, Eygeris Y, Ryals RC, Jozić A (2024). G Sahay Nat Nanotechnol.

[58] Bottini N (2013). G S Firestein Nat Rev Rheumatol.

[59] Wang Y, Han CC, Cui D, Li Y, Ma Y (2017). W Wei Int Immunopharmacol.

[60] Aihaiti Y, Song Cai Y, Tuerhong X, Ni Yang Y, Ma Y, Shi H, Zheng K, Xu P. Xu Front Pharmacol 2021,12,672054. 10.3389/fphar.2021.672054 PMC8160516 34054546

[61] Zayoud M, Marcu-Malina V, Vax E, Jacob-Hirsch J, Elad-Sfadia G, Barshack I, Kloog Y. I. Goldstein Front Immunol 2017,8,799. 10.3389/fimmu.2017.00799 PMC5500629 28736556

[62] Ohkura T, Yoshimura T, Fujisawa M, Ohara T, Marutani R, Usami K. A. Matsukawa Front Immunol 2019,10,17. 10.3389/fimmu.2019.00017 PMC6349710 30723473

[63] Linnenberger R, Hoppstädter J, Wrublewsky S, Ampofo E. ,A. K. Kiemer Int J Mol Sci 2021,22. 10.3390/ijms222212480 PMC8623589 34830364

[64] Baumann D, Drebant J, Hägele T, Burger L, Serger C, Lauenstein C, Dudys P, Erdmann G. R Offringa J Immunother Cancer 2021,9. 10.1136/jitc-2020-002319 PMC8292803 34285105

[65] Fukui S, Iwamoto N, Takatani A, Igawa T, Shimizu T, Umeda M, Nishino A, Horai Y, Hirai Y, Koga T, Kawashiri SY, Tamai M, Ichinose K, Nakamura H, Origuchi T, Masuyama R. K. Kosai, K. Yanagihara,A. Kawakami Front Immunol 2017,8,1958. 10.3389/fimmu.2017.01958 PMC5766997 29375576

[66] Li J, Chen L, Xu X, Fan Y, Xue X, Shen M. X. Shi Small 2020,16,e2005661. 10.1002/smll.202005661 33205596

[67] Jia N, Gao Y, Li M, Liang Y, Li Y, Lin Y, Huang S, Lin Q, Sun X, He Q, Yao Y, Zhang B, Zhang Z. L. Zhang Signal Transduct Target Ther 2023,8,280. 10.1038/s41392-023-01499-0 PMC10374631 37500654

[68] Chen Y, Li J, Ma B, Li N, Wang S, Sun Z, Xue C, Han Q, Wei J, Zhao RC (2020). Aging.

[69] Chang L, Kan L (2021). J Inflamm Res.

[70] Xu XL, Li WS, Wang XJ, Du YL, Kang XQ, Hu JB, Li SJ, Ying XY (2018). J You Y Z Du Nanoscale.

[71] Wang X, Chen S, Lu R, Sun Y, Song T, Nie Z, Yu C. Y. Gao Heliyon 2022,8,e11495. 10.1016/j.heliyon.2022.e11495 PMC9668683 36406687

[72] Yu Q, Wang D, Wen X, Tang X, Qi D, He J, Zhao Y, Deng W. T. Zhu Am J Physiol Lung Cell Mol Physiol 2020,L318723–741. 10.1152/ajplung.00255.2019 PMC7191475 32073873

[73] Agrawal AK, Aqil F, Jeyabalan J, Spencer WA, Beck J, Gachuki BW, Alhakeem SS, Oben K, Munagala R, Bondada S (2017). R C Gupta Nanomed.

[74] Munagala R, Aqil F, Jeyabalan J (2016). R C Gupta Cancer Lett.

[75] Aqil F, Kausar H, Agrawal AK, Jeyabalan J, Kyakulaga AH, Munagala R (2016). R Gupta Exp Mol Pathol.

[76] Dong M, Wu S, Xu H, Yu X, Wang L, Bai H. W. Niu Front Bioeng Biotechnol 2021,9,615920. 10.3389/fbioe.2021.615920 PMC7952636 33718337

[77] Xu R, Bai Y, Min S, Xu X, Tang T (2020). S Ju Int J Nanomed.

[78] Hu J, Jiang Y, Wu X, Wu Z, Qin J, Zhao Z, Li B, Xu Z, Lu X, Wang X. X. Liu Stem Cell Res Ther 2022,13349. 10.1186/s13287-022-03037-1 PMC9327292 35883151

[79] Wang Q, Ye Q, Xi X, Cao X, Wang X, Zhang M, Xu Y, Deng T, Deng X, Zhang G. C. Xiao Front Immunol 2023,14,1135014. 10.3389/fimmu.2023.1135014 PMC10040599 36993980

[80] Mukai T, Gallant R, Ishida S, Kittaka M, Yoshitaka T, Fox DA, Morita Y, Nishida K, Rottapel R. Y. Ueki Arthritis Rheumatol 2015,67,656 – 67. 10.1002/art.38975 PMC4342302 25470448

[81] Shu H, Zhao H, Shi Y, Lu C, Li L, Zhao N, Lu A. X. He Chin Med 2021,16,31. 10.1186/s13020-021-00439-w PMC8042720 33845855

[82] Ekinci Akdemir FN, Albayrak M, Çalik M, Bayir Y. İ. Gülçin Biomedicines 2017,5. 10.3390/biomedicines5020018 PMC5489804 28536361

